# *PaACL* silencing accelerates flower senescence and changes the proteome to maintain metabolic homeostasis in *Petunia hybrida*

**DOI:** 10.1093/jxb/eraa208

**Published:** 2020-05-04

**Authors:** Huina Zhao, Shiwei Zhong, Lina Sang, Xinyou Zhang, Zeyu Chen, Qian Wei’s, Guoju Chen, Juanxu Liu, Yixun Yu

**Affiliations:** 1 College of Horticulture, South China Agricultural University, Guangzhou, China; 2 Guangdong Key Laboratory for Innovative Development and Utilization of Forest Plant Germplasm, College of Forestry and Landscape Architecture, South China Agricultural University, Guangzhou, China; 3 Lingnan Guangdong Laboratory of Modern Agriculture, Guangzhou, China; 4 University College Dublin, Ireland

**Keywords:** Acetyl-CoA, acetylome, ATP-citrate lyase, metabolic homeostasis, petunia, proteome

## Abstract

Cytosolic acetyl-CoA is an intermediate of the synthesis of most secondary metabolites and the source of acetyl for protein acetylation. The formation of cytosolic acetyl-CoA from citrate is catalysed by ATP-citrate lyase (ACL). However, the function of ACL in global metabolite synthesis and global protein acetylation is not well known. Here, four genes, *PaACLA1*, *PaACLA2*, *PaACLB1*, and *PaACLB2*, which encode the ACLA and ACLB subunits of ACL in *Petunia axillaris*, were identified as the same sequences in *Petunia hybrida* ‘Ultra’. Silencing of *PaACLA1-A2* and *PaACLB1-B2* led to abnormal leaf and flower development, reduced total anthocyanin content, and accelerated flower senescence in petunia ‘Ultra’. Metabolome and acetylome analysis revealed that *PaACLB1-B2* silencing increased the content of many downstream metabolites of acetyl-CoA metabolism and the levels of acetylation of many proteins in petunia corollas. Mechanistically, the metabolic stress induced by reduction of acetyl-CoA in *PaACL*-silenced petunia corollas caused global and specific changes in the transcriptome, the proteome, and the acetylome, with the effect of maintaining metabolic homeostasis. In addition, the global proteome and acetylome were negatively correlated under acetyl-CoA deficiency. Together, our results suggest that ACL acts as an important metabolic regulator that maintains metabolic homeostasis by promoting changes in the transcriptome, proteome. and acetylome.

## Teaser

*PaACL* silencing reduced anthocyanin contents, accelerated flower senescence, and changed the proteome and acetylome to maintain metabolic homeostasis in petunia

## Introduction

Acetyl-coenzyme A (CoA) is the intermediate precursor for the biosynthesis of a wide variety of phytochemicals, including carbohydrates, malonyl-CoA, organic acids, fatty acids, isoprenoids, flavonoids, and stilbenoids (Fatland *et al.*, 2002; Oliver *et al.*, 2009; Finkemeier *et al.*, 2011; Wu *et al.*, 2011; Chypre *et al.*, 2012; Xing and Poirier, 2012). Acetyl-CoA is found in the cytosol and in organelles, and is membrane impermeable. Therefore, acetyl-CoA biogenesis is thought to occur in each subcellular compartment where it is required, except for the nucleus, into which cytosolic acetyl-CoA most likely diffuses via the nuclear pore (Brooks and Stumpf, 1966; Fatland *et al.*, 2002; Chen *et al.*, 2017a). Cytosolic acetyl-CoA is also the source of acetyl for protein acetylation, including histone acetylation (Chen *et al.*, 2017a). In yeast and mammalian cells, the levels of acetylation of histone H3 and H4 were affected by cellular acetyl-CoA levels (Takahashi *et al.*, 2006; Wellen *et al.*, 2009; Cai *et al.*, 2011; Galdieri and Vancura, 2012; Lee *et al.*, 2014). In the plant *Arabidopsis thaliana*, the increase in H3K27 acetylation (H3K27ac) is dependent on cytoplasmic ATP-citrate lyase (ACL), and H3K27ac is an important link between the cytosolic level of acetyl-CoA and gene expression in response to the dynamic metabolic environments in the plant (Chen *et al.*, 2017a).

The formation of cytosolic acetyl-CoA from citrate is catalysed by ACL (Aoshima, 2007). ACL has been characterized in plants at the genomic level; it is a heteropolymer composed of ACLA and ACLB subunits located in the cytoplasm, implying that it generates a cytosolic pool of acetyl-CoA (Fatland *et al.*, 2002). In *A. thaliana*, the ACLA subunit is encoded by three genes and the ACLB subunit is encoded by two genes (Fatland *et al.*, 2002). ACL is involved in plant growth and development, secondary metabolism, and stress. In *A. thaliana*, moderately reduced ACL activity resulted in miniaturized organs, smaller cells, aberrant plastid morphology, and reduced cuticular wax deposition in vegetative tissue (Fatland *et al.*, 2002). Overexpression of AtACL can promote the synthesis of wax, cutin, and rubber, revealing the important position of ACL in controlling the direction of carbon flow through cytoplasmic acetyl-CoA (Xing *et al.*, 2014). Overexpression of sugarcane (*Saccharum officinarum*) SoACLA-1 in tobacco enhanced its drought resistance (Phan *et al.*, 2016). In canola (*Brassica napus*), unchanged ACL protein might maintain the activity of the tricarboxylic acid cycle for adaptation to osmotic stress in leaves of drought-tolerant canola inoculated with *Enterobacter* sp. S16-3 as plant growth-promoting bacteria (Kazemi *et al.*, 2017). The expression of ACL in *Raphanus sativus* roots was up-regulated under heavy metal stress (Wang *et al.*, 2016). After treatment with low light, strong light, water stress, and abscisic acid, the expression level of *ACL* increased over a certain period (Tong, 2009). However, the function of ACL in global metabolite synthesis and global protein acetylation in plants is not known.

Petunia (*Petunia hybrida*) is a good system for research on plant growth and development (Gerats and Vandenbussche, 2005). In this study, we found that both the ACLA and ACLB subunits were encoded by two genes in petunia. Silencing of any single *PaACL* gene did not result in a visible phenotypic change, whereas silencing of both *PaACLA1* and *PaACLA2* (*PaACLA1-A2*) or both *PaACLB1* and *PaACLB2* (*PaACLB1-B2*) resulted in a similar phenotype, comprising abnormal development of leaves and flowers, decreased total anthocyanin content, increased chlorophyll content, acceleration of flower senescence, and increased ethylene production. Metabolomic analysis of petunia revealed that *PaACLB1-B2* silencing changed the metabolite profile and that most metabolites, including flavonoids, were up-regulated. Mechanistically, the metabolic stress induced by the reduction of acetyl-CoA in *PaACL*-silenced petunia corollas resulted in global and specific changes in the transcriptome, the proteome, and the acetylome to maintain metabolic homeostasis.

## Materials and methods

### Plant materials and growth conditions

*Petunia hybrida* ‘Ultra’ plants were grown under greenhouse conditions (23±2 °C, 14 h light/10 h dark) (Yang *et al.*, 2017). The roots, stems, and leaves were collected from plants in the vegetative stage when the height of the plants was approximately 25 cm. The flowers were harvested at anthesis (corollas 90° reflexed) and immediately placed in tap water. All tissues were frozen in liquid nitrogen and stored at –80 °C until use. All experiments were conducted at least three times with independently collected and extracted tissues unless otherwise noted.

### RNA extraction, RT–PCR, and cloning of the petunia *ACL* genes

Total RNA was isolated according to the methods of Liu *et al.* (2011). A commercially available kit (TSK314s, Tsingke Guangzhou, China) was used for reverse transcription of petunia mRNA. Full-length *PaACLA1* (Peaxi162Scf00357g00428.1), *PaACLA2* (Peaxi162Scf00158g00011.1), *PaACLB1* (Peaxi162Scf00228g00327.1), and *PaACLB2* (Peaxi162Scf01160g00016.1) cDNA was isolated from *P. hybrida* ‘Ultra’ using specific primers (see Supplementary Table S1 at *JXB* online) based on their sequences in the *Petunia axillaris* genome (https://solgenomics.net/organism/Petunia_axillaris/genome).

### Sequence analysis

Alignments were conducted, and a phylogenetic tree was generated using DNAMAN software. An identity search for nucleotides and translated amino acids was conducted using the National Center for Biotechnology Information (NCBI) BLAST network server (https://blast.ncbi.nlm.nih.gov/Blast.cgi).

### Quantitative real-time PCR assays

Quantitative real-time PCR (qPCR) assays were performed according to previous methods (Liu *et al.*, 2011). Analyses were conducted following the Minimum Information for Publication of Quantitative Real-Time PCR Experiments guidelines (Bustin *et al.*, 2009; Tan *et al.*, 2014). *Cyclophilin* (*CYP*) (accession no. EST883944) and *Actin* (accession no. FN014209) were used as the internal reference genes to quantify cDNA abundance (Mallona *et al.*, 2010; Yang *et al.*, 2017). Similar results were obtained with both reference genes. The data reported in the Results represent relative expression values calculated by the 2^−ΔΔC^^t^ approximation method (Livak and Schmittgen, 2001). The sequences of all primers used for qPCR analysis are listed in Supplementary Table S2. Three biological replicates, each including three technical repeats, were analysed for each treatment.

### Agroinoculation of pTRV vectors

pTRV2-PaACLA1, pTRV2-PaACLA2, pTRV2-PaACLB1, pTRV2-PaACLB2, pTRV2-PaACLA1-PaACLA2, and pTRV2-PaACLB1-PaACLB2 vectors were constructed by amplifying the gene sequences of approximately 250 bp of the 3′ untranslated region of the four *PaACL* genes using the specific primers listed in Supplementary Table S3 and cloning them into the pTRV2 vector. *Agrobacterium tumefaciens* (strain GV3101) transformed with pTRV1 and pTRV2 derivatives were prepared as previously described (Spitzer-Rimon *et al.*, 2012; Tan *et al.*, 2014). Approximately 30 petunia plants were inoculated with each vector for virus-induced gene silencing (VIGS) experiments. The inoculated plants were grown under greenhouse conditions.

### Determination of ACL activity

Crude protein extracts were desalted by chromatography through Sephadex G-25 (Sigma-Aldrich, St. Louis, MO, USA), and ACL activity was determined using a coupled spectrophotometric assay (Fatland *et al.*, 2002).

### Pigment measurement

Four or five mature petunia leaves were collected from each plant and freeze-dried, and three replicate methanol extractions were prepared for each type of plant using the method of Wellburn (1994). The absorbance of the solution was read with a spectrophotometer at 646.8 nm, 663.2 nm, and 470.0 nm against the solvent (acetone) blank. The individual concentrations of total chlorophyll and total carotenoids were calculated from the spectrophotometric measurements (Lichtenthaler, 1987).

Anthocyanin extraction and analysis were performed as previously described (Chen *et al.*, 2017b). Petunia flowers were harvested at anthesis and the corolla limbs were collected. Three biological replicates were analysed for each treatment.

### Acetyl-CoA measurement

Approximately 1 g of corollas was ground in the presence of liquid nitrogen, and 9 ml of phosphate-buffered saline was then added. The sample was thoroughly vortexed and incubated for 20 min at room temperature. The mixture was centrifuged at 16 873 *g* for 15 min to separate the layers, and the supernatant was collected. The content of acetyl-CoA in the supernatant was determined using a kit (JN709212, Ji-Ning Bio-tech, Shanghai, China) according to the manufacturer’s instructions.

### Measurement of ethylene

Ethylene measurements were performed according to the method of Tan *et al.* (2014). Flowers were collected and placed individually in 150 ml airtight containers. The containers were capped and incubated at 25 °C for 1 h. Then, 2 µl of head-space gas was withdrawn using a gas-tight hypodermic syringe and injected into a gas chromatograph (GC 17A, Shimadzu, Kyoto, Japan) for measurement of ethylene concentration. The gas chromatograph was equipped with a flame ionization detector and an activated alumina column. All measurements were performed with five replicates.

### Scanning electron microscopy

The mature leaves and corolla limbs from petunia plants were cut into 3–5 mm^2^ pieces. The samples were fixed, dehydrated, and coated with gold by using ion sputtering equipment, according to our previously described protocol (Yang *et al.*, 2017). Samples were observed with a scanning electron microscope (XL-30-ESEM, FEI, The Netherlands) at 10 kV acceleration and photographed.

### Analysis of widely targeted metabolites

The collected petunia corollas were freeze-dried and ground to powder, and 0.1 g of powder was placed in 1.0 ml of 70% aqueous methanol at 4 °C and stored overnight. Then, the extract was centrifuged at 10 000 *g* for 10 min, and the supernatant was filtered through a microporous membrane (0.22 μm pore size) for analysis.

The metabolites were analysed by ultra-performance liquid chromatography (UPLC) (Shim-pack UFLC SHIMADZU CBM30A, http://www.shimadzu.com.cn/) and tandem mass spectrometry (MS/MS) (AB SCIEX 6500 QTRAP) under the conditions described by Li and Song (2019). In the triple quadrupole, each ion pair was scanned for detection based on the optimized decompression potential and collision energy (Chen *et al.*, 2013). The qualitative and quantitative MS analysis of metabolites in the samples was performed on metabolites based on the self-built Metware database (MWDB) (Metware, Wuhan, China), and multiple reaction monitoring (MRM) (Chen *et al.*, 2013).

### Transcriptomic analysis of corollas by RNA sequencing

Corollas of *PaACLB1-B2* silenced petunia lines and controls were collected for mRNA library construction and sequencing as described before (Guo *et al.*, 2017). Corolla RNA was isolated using TRIzol reagent (Promega, USA) according to the manufacturer’s protocol. The extracted RNA was quantified and its integrity was measured on a 1.0% denaturing agarose gel. The poly(A)-containing mRNA molecules were purified using Sera-mag Magnetic Oligo(dT) Beads (Thermo Scientific) and were fragmented by incubation in RNA fragmentation reagent (Ambion). The fragmented mRNA was then converted into double-stranded cDNA using a SuperScript double-stranded cDNA synthesis kit (Invitrogen) by priming with random hexamers. Strand non-specific transcriptome libraries were prepared using the NEBNext mRNA Library Prep Reagent Set (New England Biolabs). These cDNA libraries were amplified and sequenced on an Illumina HiSeq platform (Metware, Wuhan, China). Raw reads, including the adaptor sequences, low-quality sequences, and unknown nucleotides, were filtered into clean reads using the standard quality control technique. Clean reads were aligned to petunia reference genome sequences (https://solgenomics.net/organism/Petunia_axillaris/genome) by hierarchical indexing for spliced alignment of transcripts in the HISAT2 application. The fragments per kilobase of transcript per million reads mapped (FPKM) method was used to calculate normalized expression levels. Differentially expressed genes (DEGs) between *PaACLB1-B2* silenced samples and controls were identified by the NOISeq method according to the default criterion of a 2-fold change (*P*-value <0.05).

### Protein extraction and trypsin digestion

Protein extraction and trypsin digestion were performed according our previous protocol (Guo *et al.*, 2017). The corollas (~2.5 g for each sample) were ground to a powder in liquid nitrogen and then transferred to a 5 ml centrifuge tube. A volume of lysis buffer (8 M urea, 1% Triton-100, 10 mM dithiothreitol, and 1% protease inhibitor cocktail) four times the volume of the sample powder was added, followed by sonication three times on ice using a high-intensity ultrasonic processor (Scientz). The remaining debris was removed by centrifugation at 20 000 *g* at 4 °C for 10 min. Finally, the protein was precipitated with cold 20% trichloroacetic acid for 2 h at –20 °C. After centrifugation at 12 000 *g* at 4 °C for 10 min, the supernatant was discarded. The remaining precipitate was washed with cold acetone three times. The protein was redissolved in 8 M urea, and the protein concentration was determined with a BCA kit according to the manufacturer’s instructions (P0011, Beyotime Biotechnology, Shanghai, China).

For digestion, the protein solution was reduced with 5 mM dithiothreitol for 30 min at 56 °C and alkylated with 11 mM iodoacetamide for 15 min at room temperature in darkness. The protein sample was then diluted by adding 100 mM triethylammonium bicarbonate (TEAB) to a urea concentration less than 2 M. Finally, trypsin was added at a 1:50 trypsin-to-protein mass ratio for the first digestion overnight, and at a 1:100 trypsin-to-protein mass ratio for a second 4 h digestion.

### TMT labelling

After trypsin digestion, the peptide was desalted in a Strata X C18 SPE column (Phenomenex) and vacuum-dried. Peptide was reconstituted in 0.5 M TEAB and processed according to the manufacturer’s protocol for the tandem mass tag (TMT) kit (No. 90068, Thermo Scientific, Waltham, MA, USA). Briefly, one unit of TMT reagent was thawed and reconstituted in acetonitrile. The peptide mixtures were incubated for 2 h at room temperature and then pooled, desalted, and dried by vacuum centrifugation.

### HPLC fractionation

The tryptic peptides were fractionated by high pH reverse-phase HPLC using a Thermo Betasil C18 column (5 μm particles, 10 mm internal diameter, 250 mm length). Briefly, peptides were first separated with a gradient of 8% to 32% acetonitrile (pH 9.0) over 60 min into 60 fractions. Then, the peptides were combined into acetylation fractions and dried by vacuum centrifuging.

### Drawing of protein–metabolite co-expression networks

Protein–metabolite co-expression networks were constructed based on the Spearman correlation coefficient (Kovarik, 2013). The Spearman correlation coefficients between the quantitative values of differential proteins and differential metabolites in samples were calculated by using the cor function in R. Data with Spearman correlation coefficients greater than 0.95 or less than –0.95 between metabolites and proteins were screened out to construct the co-expression network. The network was visualized and analyzed by using cycloscape v3.7.2 software (Smoot *et al.*, 2011).

### Antibody-based acetylation peptide enrichment

To enrich acetylation peptides, tryptic peptides dissolved in NETN buffer (100 mM NaCl, 1 mM EDTA, 50 mM Tris–HCl, 0.5% NP-40, pH 8.0) were incubated with pre-washed antibody beads (lot number PTM-104, PTM Bio) at 4 °C overnight with gentle shaking. Then, the beads were washed four times with NETN buffer and twice with water. The bound peptides were eluted from the beads with 0.1% trifluoroacetic acid. Finally, the eluted fractions were combined and vacuum-dried. For LC-MS/MS analysis, the resulting peptides were desalted with C18 ZipTips (Millipore) according to the manufacturer’s instructions.

### LC-MS/MS analysis

LC-MS/MS analysis was performed according to a previously described protocol (Guo *et al.*, 2017). The tryptic peptides were dissolved in 0.1% formic acid (solvent A) and directly loaded on to a homemade reversed-phase analytical column (15 cm length, 75 μm internal diameter). The gradient consisted of an increase from 6% to 23% of solvent B (0.1% formic acid in 98% acetonitrile) over 26 min, followed by an increase from 23% to 35% in 8 min, then climbing to 80% in 3 min and then holding at 80% for the last 3 min, all at a constant flow rate of 400 nl min^–1^, on an EASY-nLC 1000 UPLC system.

The peptides were subjected to nanospray ionization followed by MS/MS in Q Exactive^TM^ Plus (Thermo Fisher Scientific) coupled online to the UPLC. The electrospray voltage applied was 2.0 kV. The m/z scan range was 350 to 1800 for full scan, and intact peptides were detected in the Orbitrap (Thermo Fisher Scientific) at a resolution of 70 000. Peptides were then selected for MS/MS using the normalized collision energy setting at 28, and the fragments were detected in the Orbitrap at a resolution of 17 500. A data-dependent procedure was used that alternated between one MS scan and 20 MS/MS scans with 15.0 s dynamic exclusion. Automatic gain control was set at 5E4. The fixed first mass was set at 100 m/z.

### Database search

The MS/MS data were processed using a Maxquant search engine (v.1.5.2.8). Tandem mass spectra were searched against the petunia database (https://solgenomics.net/organism/Petunia_axillaris/genome) concatenated with a reverse decoy database. Trypsin/P was specified as a cleavage enzyme allowing up to four missing cleavages. The mass tolerance for precursor ions was set at 20 ppm in the first search and 5 ppm in the main search, and the mass tolerance for fragment ions was set at 0.02 Da. Carbamidomethyl on Cys was specified as a fixed modification, and acetylation modification and oxidation on Met were specified as variable modifications. The false discovery rate (FDR) was adjusted to <1%, and the minimum score for modified peptides was set to >40.

### Subcellular localization analysis

We used WoLF PSORT (v0.2, http://www.genscript.com/psort/wolf_psort.html), a subcellular localization predication software program, to predict the subcellular localization of proteins (Horton *et al.*, 2007). WoLF PSORT is an updated version of PSORT/PSORT II for the prediction of eukaryotic sequences.

### Gene ontology enrichment and pathway analysis

Proteins were classified by gene ontology (GO) annotation into three categories: biological process, cellular compartment, and molecular function. For each category, a two-tailed Fisher’s exact test was used to test the enrichment of the differentially modified protein against all identified proteins. GO annotations with a corrected *P*-value <0.05 were considered significant.

The KEGG database (v.2.5, http://www.kegg.jp/kegg/mapper.html) was used to identify enriched pathways by a two-tailed Fisher’s exact test to test the enrichment of the differentially modified protein against all identified proteins (Kanehisa and Goto, 2000). Pathways with a corrected *P*-value <0.05 were considered significant. These pathways were classified into hierarchical categories according to the KEGG website.

### Motif analysis

The software Motif-x was used to analyse the model of sequences constituted with amino acids in specific positions of modify-21-mers (10 amino acids upstream and downstream of the site) in all protein sequences. All of the database protein sequences were used as background database parameters and other default parameters.

### Preparation of specific antibodies and western blot analysis

Western blot analyses were performed according to the methods of Liu *et al.* (2019). The peptides (Supplementary Table S4) expressed in *Escherichia coli* of three proteins, PamCS (Peaxi162Scf00402g00612.1), PaPDC2 (pyruvate decarboxylase-2, Peaxi162Scf00014g00030.1) and PaGELP (GDSL, esterase/lipase, Peaxi162Scf00038g01135.1), and the synthetic acetylation peptides (QQFNKacKMNS) of the protein PaGELP255Kac, were used as antigens for antibody production in rabbits from Diaan (http://www.dia-an.cn/). These antibodies were used for blotting analysis (Liu *et al.* 2019).

### Statistical analysis

Statistical analysis was performed using one-way analysis of variance followed by Duncan’s multiple range test with at least three replicates. *P*-values ≤0.05 were considered significant.

## Results

### Identification and expression analysis of the *ACL* gene family in *Petunia hybrida*

To identify petunia *ACL* genes, we used the cDNA sequences of *AtACLA1* (AY113979) and *AtACLB1* (AY050858) from *A. thaliana* (Fatland *et al.*, 2002) as a query in BLAST and searched the *P. axillaris* draft genome sequence v1.6.2. Sequences from two *ACLA*s, *PaACLA1* and *PaACLA2*, and two *ACLB*s, *PaACLB1* and *PaACLB2*, of *P. axillaris* were recovered. Using the special primers of these gene sequences, we obtained the same full-length sequences of these genes from *P. hybrida* ‘Ultra’ by RT–PCR. The results of multiple sequence alignment showed that the four *PaACL*s had high identity to their homologs in *A. thaliana* and *Solanum lycopersicum* (Supplementary Fig. S1).

**Fig. 1.**
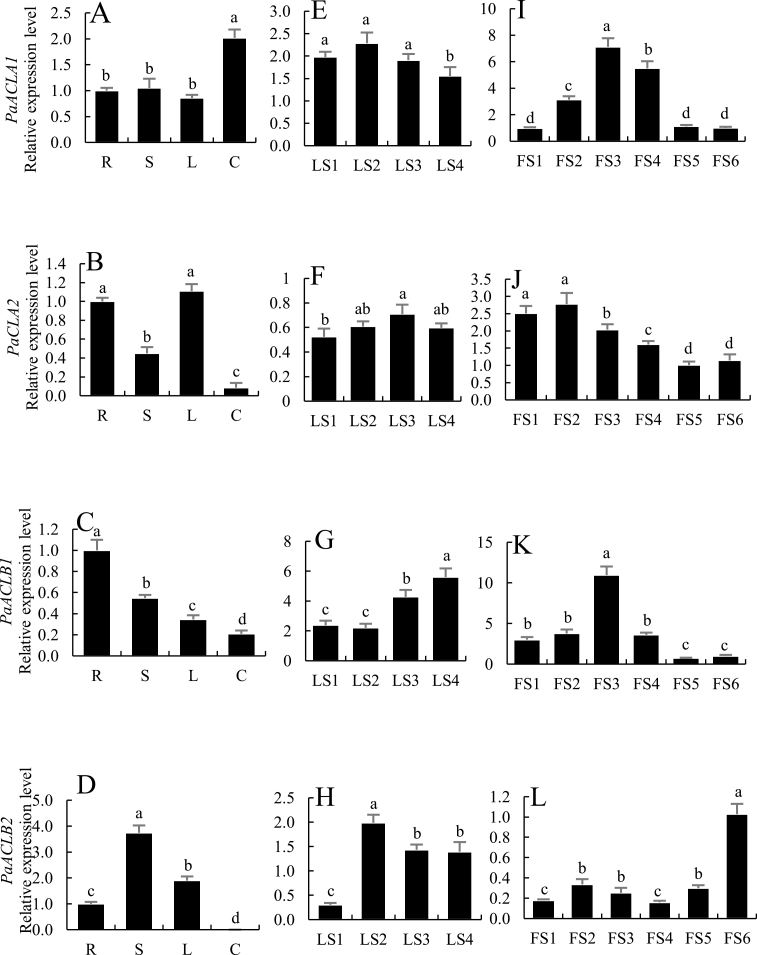
Expression patterns of *PaACLA*s and *PaACLB*s determined using quantitative real-time PCR. (A–D) Expression of *PaACLA1* (A), *PaACLA2* (B), *PaACLB1* (C), and *PaACLB2* (D) in different organs. R, roots; S, stems; L, leaves; C, corollas. (E–H) Expression of *PaACLA1* (E), *PaACLA2* (F), *PaACLB1* (G), and *PaACLB2* (H) during leaf development. LS1, young leaves (1 cm); LS2, growing leaves (3 cm); LS3, mature leaves (4 cm); LS4, old leaves. (I–L) Expression of *PaACLA1* (I), *PaACLA2* (J), *PaACLB1* (K), and *PaACLB2* (L) during flower development. Flower development stages: FS1 (0.5 cm in length), FS2 (1.0 cm), FS3 (2.0 cm), FS4 (3.0 cm), FS5 (4.0 cm), and FS6 (anthesis). *Cyclophilin* (accession no. EST883944) was used as the internal reference gene to quantify cDNA abundance. Data are presented as means ±SD (*n*=3). Different letters indicate significant differences (*P*≤0.05).

Four *PaACL* transcriptional levels were examined in different plant organs and during leaf and bud development by qPCR using *CYP* and *Actin* as the internal reference genes. *PaACLA1*, *PaACLA2*, *PaACLB1*, and *PaACLB2* transcripts were all detected in roots, stems, leaves, and corollas. The expression levels of *PaACLA1*, *PaACLA2*, *PaACLB1* and *PaACLB2* were strongest in the corollas, leaves, roots, and stems, respectively. The lowest expression of *PaACLA1* was in leaves, and the expression of the other three genes was lowest in flowers (Fig. 1A–D; Supplementary Fig. S2A–D).

To further assess the expression of *PaACLA*s and *PaACLB*s during leaf development, leaf development was divided into four stages: LS1 (young leaves, 1.0 cm in length), LS2 (growing leaves, 3.0 cm), LS3 (mature leaves, 4.0 cm), and LS4 (old leaves). As shown in Fig. 1E–H and Supplementary Fig. S2E–H, the expression of *PaACLA1* did not change significantly from stage LS1 to LS3, whereas the expression of *PaACLA2* increased significantly from LS1 to LS3. The expression of *PaACLB1* increased gradually from stage LS2 to LS4. The expression of *PaACLB2* significantly increased from stage LS1 to LS2 and then decreased.

Flower development was divided into six stages: FS1 (0.5 cm in length), FS2 (1.0 cm), FS3 (2.0 cm), FS4 (3.0 cm), FS5 (4.0 cm), and FS6 (anthesis). As shown in Fig. 1I–L and Supplementary Fig. S2I–L, the expression of *PaACLA1* significantly increased from stage FS1 to FS3 and then decreased, the expression of *PaACLA2* decreased gradually from FS1 to FS5, the expression of *PaACLB1* significantly increased from FS2 to FS3 and then decreased, and the expression of *PaACLB2* remained fairly stable from FS1 to FS5 and then increased.

### VIGS-mediated *PaACLA1-A2* and *PaACLB1-B2* silencing leads to abnormal leaf and flower development and reduces total anthocyanin content

**Fig. 2.**
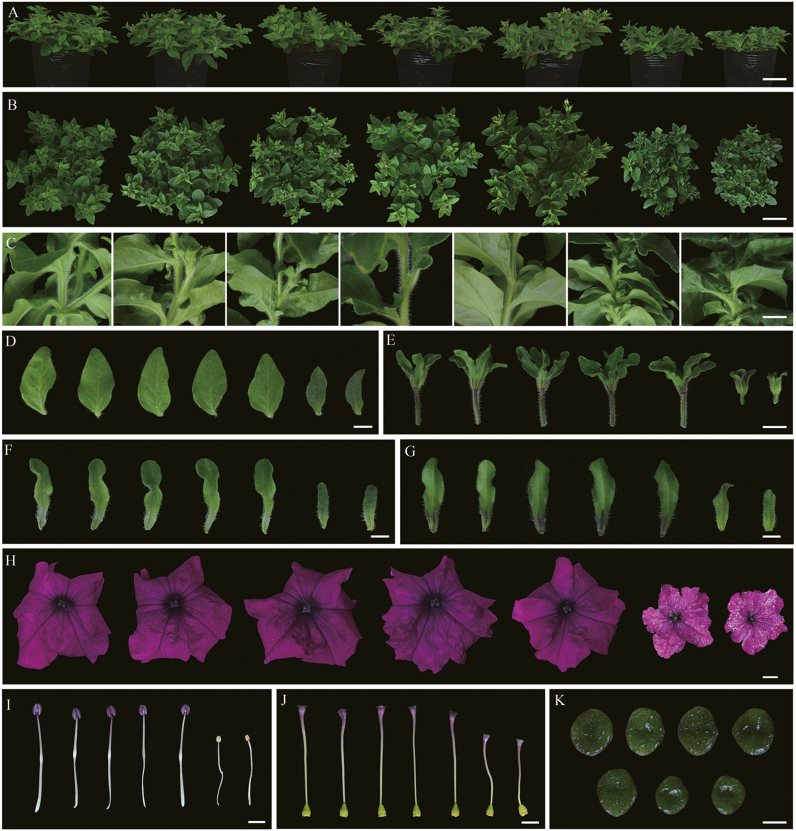
Phenotypic alterations caused by VIGS-mediated silencing of *PaACLA*s and *PaACLB*s in petunia plants. (A–G) Left to right: 5-week-old pTRV2-treated (control) and *PaACLA1*-, *PaACLA2*-, *PaACLB1*-, *PaACLB2*-, *PaACLA1-A2*-, and *PaACLB1-B2*-silenced plants: side view (A), top view (B), stems (C), leaves (D), pedicels (E), abaxial surface of sepals (F), and adaxial surface of sepals (H). (H) Left to right: flowers of the control and *PaACLA1*-, *PaACLA2*-, *PaACLB1*-, *PaACLB2*-, *PaACLA1-A2*-, and *PaACLB1-B2*-silenced plants. (I–K) Left to right: control and *PaACLA1*-, *PaACLA2*-, *PaACLB1*-, *PaACLB2*-, *PaACLA1-A2*- and *PaACLB1-B2*-silenced stamens (I), pistils (J), and stigmas (K) from 5-week-old plants. Bars=4 cm in (A) and (B); 1 cm in (C), (E), and (H); 0.5 cm in (D), (F), (G), (I), and (J); 0.15 cm in (K).

**Fig. 3.**
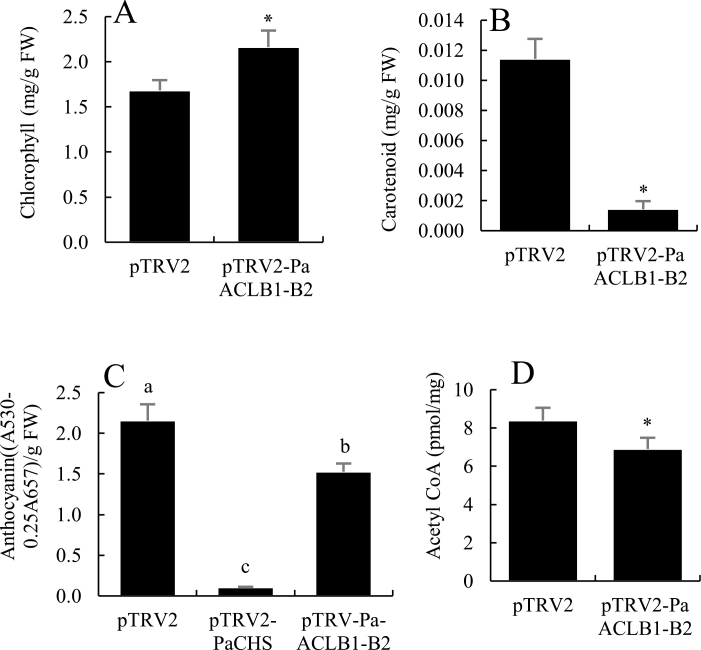
Effects of *PaACLB1-B2* silencing on the chlorophyll, carotenoid, anthocyanin, and acetyl-CoA content in petunia. (A) Chlorophyll and (B) carotenoid content in leaves. (C) Anthocyanin content in corollas. (D) Acetyl-CoA content in corollas. Data are presented as means ±SD (*n*=3). *P≤0.05.

To identify the function of the *PaACLA1-A2* and *PaACLB1-B2* genes, vectors were constructed to suppress the expression of these genes because we had already established a highly efficient VIGS system in petunia ‘Ultra’ (Tan *et al.*, 2014; Liu *et al*., 2017). Approximately 30 days after petunia seedlings were infected, no visible change was observed in any single-gene-silenced plants compared with the controls. By contrast, pTRV2-PaACLA1-A2- and pTRV2-PaACLB1-B2-infected plants had similar abnormal phenotypes, with decreased plant height, internodes, and crown width (Supplementary Table S5; Fig. 2A–C; Supplementary Fig. S3A–C). The plants were small, and the leaves were dark green, small, and uneven in shape (Fig. 2A–D; Supplementary Fig. S3A–D). The chlorophyll content was significantly higher and the carotenoid content was significantly lower in leaves, compared with controls (Fig. 3A). The flowers were smaller and the floral colour was lighter than controls, and there were some cracked and necrotic tissues at the edge of the corollas in the *PaACLA1-A2*- and *PaACLB1-B2*-silenced plants (Fig. 2H; Supplementary Fig. S3E–H). The pedicels, filaments, and styles of *PaACLB1-B2*-silenced plants were short, and the stigmas were small (Supplementary Table S5; Fig. 2E). The sepals were smaller and had a deeper colour than the controls (Fig. 2F, G). The colour of the anthers, the top of the pedicels, and the base of the sepals was lighter than that of the control (Fig. 2I, J). Little or no pollen was found in the anthers of *PaACLB1-B2*-silenced plants (Supplementary Fig. S3I). Self-pollination yielded hardly any seeds, while pollination with normal pollen yielded more seeds than self-pollination but fewer seeds than the controls (168.80±11.84 for *PaACLB1-B2-*silenced plants pollinated with normal pollen versus 221.80±15.83 for controls; *P*<0.05). The seeds produced by cross-pollinated *PaACLB1-B2*-silenced plants were lighter in colour than control seeds (Supplementary Fig. S3J). The total content of anthocyanins in *PaACLB1-B2*-silenced corollas was significantly lower compared than that of control corollas, but higher than that of *PaCHS*-silenced flowers, used as a positive control (Fig. 3B). In addition, the content of acetyl-CoA in *PaACLB1-B2*-silenced corollas was significantly lower compared with the control (Fig. 3C).

We examined the expression of *PaACLA*s and *PaACLB*s in corollas of different silenced plants by qPCR using *CYP* and *Actin* as internal reference genes (Supplementary Figs S4 and S5). As expected, treatment with pTRV2-PaACLA1, pTRV2-PaACLA2, pTRV2-PaACLB1, and pTRV2-PaACLB2 significantly reduced the expression of *PaACLA1*, *PaACLA2*, *PaACLB1*, and *PaACLB2*, respectively. pTRV2-PaACLA1-A2 and pTRV2-PaACLB1-B2 treatment significantly reduced the expression of the two *PaACLA*s and the two *PaACLB*s, respectively, but did not significantly change the expression of *PaACLB*s and *PaACLA*s, respectively (Supplementary Figs S4 and S5).

The ACL activities in leaves and corollas treated with pTRV2-PaACLB1-B2 and pTRV2 were determined using the purified proteins and a coupled spectrophotometric assay (Fatland *et al.*, 2002). The results showed that *PaACLB1-B2* silencing significantly reduced ACL activities in corollas compared with the control (Supplementary Fig. S6).

### *PaACLB1-B2* silencing changes the shape and size of cells

**Fig. 4.**
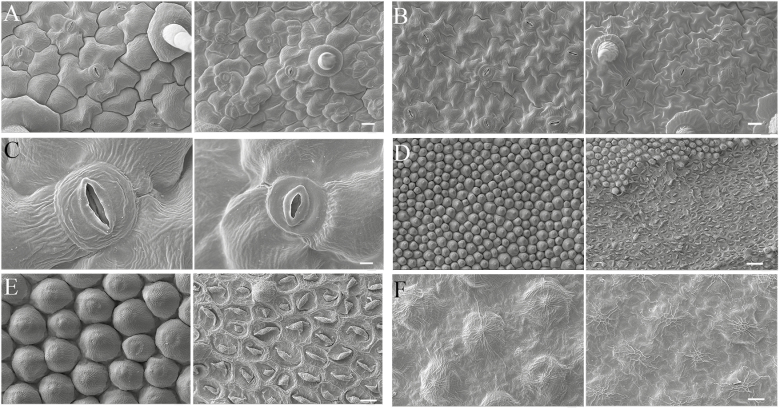
Scanning electron micrographs of the leaves of *PaACLB1-B2*-silenced plants (left panels) compared with those of control plants (right panels). (A) Leaf abaxial epidermal cells. (B) Leaf adaxial epidermal cells. (C) Guard cells of leaf abaxial surface. (D, E) Petal abaxial epidermal cells. (F) Petal adaxial epidermal cells. Bars=20 µm in (A) and (B); 4 µm in (C); 40 µm in (D); 10 µm in (E) and (F).

Scanning electron micrographs showed that the size of leaf and corolla abaxial and adaxial epidermis cells was smaller in the *PaACLB1-B2*-silenced plants than in controls (Supplementary Table S5; Fig. 4). In addition, there were a large number of dead cells in necrotic corolla tissue (Supplementary Table S5; Fig. 4D, E).

### *PaACLB1-B2* silencing accelerates flower senescence and increases ethylene production

**Fig. 5.**
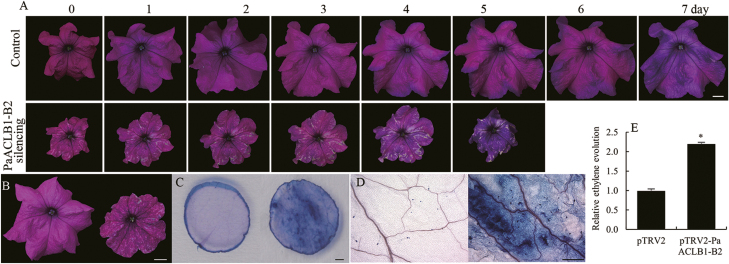
*PaACLB1-B2* silencing accelerates flower senescence in petunia. (A) Flowers of *PaACLB1-B2*-silenced and control plants. (B) Corollas for trypan blue dyeing. (C, D) Corolla tissues after trypan blue dyeing. (E) Ethylene production in *PaACLB1-B2*-silenced and control corollas. *P≤0.05. Bars=1 cm in (A) and (B); 0.1 cm in (C); 500 μm in (D).

We found that the longevity of *PaACLB1-B2*-silenced flowers was shorter than that of control flowers (5.17±0.26 versus 7.25±0.42 days) (Fig. 5A). Trypan blue staining showed that there were more dead cells in the corollas of *PaACLB1-B2*-silenced plants than in control flowers (Fig. 5B–D). In addition, the flowers of *PaACLB1-B2*-silenced plants produced more ethylene than control flowers after being open for 3 days (Fig. 5E).

### *PaACLB1-B2* silencing changes the metabolomic profile of petunia corollas

To further analyse the effects of *PaACL* silencing on the content of the metabolites, widely targeted metabolites were detected with UPLC and MS/MS in corollas from *PaACLB1-B2*-silenced plants and controls. To ensure that the sample collected was the *PaACLB1-* and *PaACLB2*-silenced corollas, the lighter part of the corollas of plants treated with pTRV2-PaACLB1-B2 was sampled, and silencing of both *PaACLB1* and *PaACLB2* in the samples was confirmed by qPCR. The qualitative and quantitative MS analysis of metabolites in the samples was performed based on the KEGG database, MWDB database, and MRM. We identified 715 metabolites, including 182 flavonoids (Supplementary Dataset S1).

**Fig. 6.**
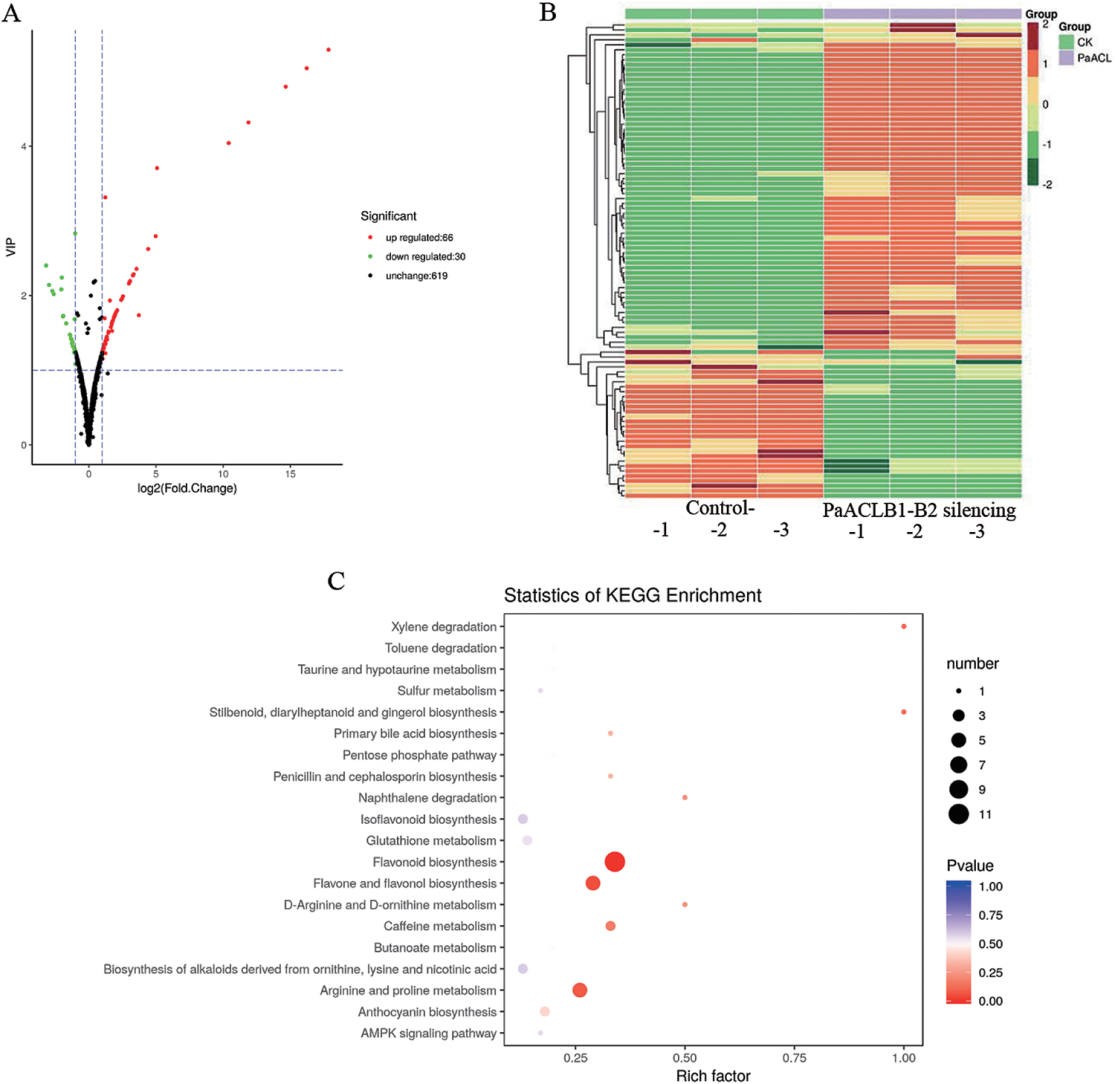
Analysis of the differential metabolites in *PaACLB1-B2*-silenced petunia corollas compared with controls. (A) Volcano plot. The green dots in the figure represent the differentially expressed metabolites that were decreased, the red dots represent the differentially expressed metabolites that were increased, and the black dots indicates metabolites for which there was no significant difference. (B) Heat map. Green indicates the differentially expressed metabolites that were decreased and red indicates the differentially expressed metabolites that were increased. The columns labelled control 1–3 and *PaACLB1-B2* silencing 1–3 refer to corolla samples from independent plants. The tree on the left side of the map indicates the relationship among the metabolites according to the normalized contents of the metabolites in different samples. The smaller the differences between metabolite contents in different samples, the closer the two metabolites are placed in the tree. (C) KEGG enrichment analysis.

Differential metabolites were screened: the criteria for screening included a fold change value of ≥2 or ≤0.5 and a variable importance in projection value of ≥1. Thirty metabolites were down-regulated and 66 were up-regulated with a high degree of repeatability (Supplementary Dataset S2; Fig. 6A, B, Supplementary Fig. S7A), and all differential metabolites were organized based on the KEGG database using FoldChange (*P*<0.05). The differential metabolites were enriched in flavonoid biosynthesis, flavone and flavonol biosynthesis, and arginine and proline metabolism in *PaACLB1-B2*-silenced corollas (Supplementary Dataset S3; Fig. 6C). Metabolites with antioxidant activity and stress-related activity, such as 17 flavonoid metabolites [including hesperetin 7-rutinoside, 3,7-Di-*O*-methylquercetin, and hesperetin 7-*O*-neohesperidoside (>30-fold)], 9 phenolamide metabolites [including *N*-caffeoyl putrescine, *N*-feruloyl tyramine, and *N*′-feruloyl putrescine (>8-fold) (Supplementary Dataset S2)], and 7 coumarin metabolites [including esculin, 6,7-dimethoxy-4-methylcoumarin, and *O*-feruloyl 3-hydroxylcoumarin (2-3-fold)], accumulated in *PaACLB1-B2*-silenced plants. In addition, two anthocyanin metabolites were significantly increased, and two anthocyanin metabolites were significantly reduced. These results are not inconsistent with the reduction of total anthocyanin content in corollas, since different anthocyanin metabolites have different content in corollas (Ando *et al.*, 1999). One terpenoid metabolite was up-regulated, and one terpenoid metabolite was down-regulated (Supplementary Dataset S2). Among the differential metabolites, all carbohydrates, coumarins, organic acids, and most differential phenolamides (9 out of 10) and lipid fatty acids (5 out of 7) were up-regulated in *PaACLB1-B2*-silenced corollas. In addition, the content of proline in *PaACLB1-B2*-silenced corollas was higher than in the control.

It is noteworthy that *PaACLB1-B2* silencing up-regulated many downstream metabolites, including 17 flavonoids, 4 organic acids, 4 lipid fatty acids, 3 lipid glycerophospholipids, and 1 terpenoid of acetyl-CoA metabolism, indicating that *PaACLB1-B2* silencing maintained metabolic homeostasis in petunia corollas.

### Transcriptome analysis reveals that *PaACLB1-B2* silencing increases the mRNA levels of genes encoding the key enzymes of biosynthesis of secondary metabolites

To explore the mechanism of *PaACL* silencing-induced up-regulation of many metabolites, we performed transcriptome analysis of corollas from *PaACLB1-B2*-silenced plants and controls. Three biological replicates were analysed for each treatment. In total, ~29.34 million clean reads were generated (Supplementary Dataset S4). *PaACLB1-B2* silencing resulted in the differential expression of 932 genes, with 733 up-regulated and 199 down-regulated genes (Supplementary Dataset S5), with a high degree of repeatability (Supplementary Fig. S7B).

To investigate the influence of the DEGs on pathways, statistical pathway enrichment analysis of *PaACLB1-B2*-silenced corollas and controls was performed based on the KEGG database using FoldChange and FDR. The DEGs were enriched in 13 KEGG metabolic pathways (Supplementary Dataset S6). The top 10 significant (*P*<0.05) metabolic pathways of the DEGs were phenylpropanoid biosynthesis; ubiquinone and other terpenoid-quinone biosynthesis; biosynthesis of secondary metabolites; metabolism of xenobiotics by cytochrome P450; glutathione metabolism; cutin, suberine, and wax biosynthesis; amino sugar and nucleotide sugar metabolism; phenylalanine metabolism; ascorbate and aldarate metabolism; and pentose and glucuronate interconversions (Supplementary Fig. S8). These results supported that acetyl-CoA is widely involved in plant secondary metabolism.

To elucidate the functional differences between the DEGs, they were analysed for GO enrichment based on clustering analysis (Supplementary Dataset S7). In the cellular component category, many of the DEGs were enriched in extracellular region, cell wall, external encapsulating structure, apoplast, intrinsic component of plasma membrane, plant-type cell wall, extracellular space, extracellular region part, plasma membrane part, and anchored component of plasma membrane. These results suggest that acetyl-CoA could be associated with the intracellular environment.

In terms of molecular functions, a large proportion of the DEGs were highly enriched in haem binding, oxidoreductase activity, monooxygenase activity, tetrapyrrole binding, iron ion binding, cofactor binding, transition metal ion binding, carbohydrate transmembrane transporter activity, hydrolase activity, and peroxidase activity. These results suggested that the reduction of acetyl-CoA could affect the activities of many enzymes, including the enzymes of stress-response and catabolic processes (Supplementary Dataset S7). The analysis of biological processes showed that many DEGs were highly enriched for the following: hormone biosynthetic process, phytosteroid metabolic process, hormone metabolic process, carbohydrate transport, regulation of hormone levels, steroid metabolic process, cellular response to chemical stimulus, hormone-mediated signalling pathway, chemical homeostasis, and steroid biosynthetic process (Supplementary Dataset S7). Significant pathway enrichment analysis showed that plant hormone biosynthesis was the most important pathway in *PaACLB1-B2* silencing and control, and plant hormone biosynthesis was the key biological event. Plant hormone biosynthesis is very important for plant growth and development, including flower senescence.

As expected, the mRNA levels of both *PaACLB1* and *PaACLB2* were down-regulated (4.5-fold) and those of both *PaACLA1* and *PaACLA2* were not significantly changed. We found that the mRNA levels of the genes encoding two key enzymes of ethylene synthesis, 1-aminocyclopropane-1-carboxylate synthase (ACS), *PaACS9* (*Peaxi162Scf00096g01846*, >100-fold) and *PaACS11* (*Peaxi162Scf00498g00034*, 29.5-fold), and 1-aminocyclopropane-1-carboxylate oxidase (ACO), *PaACO4* (*Peaxi162Scf01333g10016*, 10.2-fold) and *PaACO1* (*Peaxi162Scf00047g01927*, 3.5-fold), as well as ethylene receptor 2 (*PaETR2*, *Peaxi162Scf00024g00157*, 10-fold), were up-regulated. In addition, the expression of histone genes (*Peaxi162Scf00037g00927* and *Peaxi162Scf00697g00054*) was up-regulated (Supplementary Dataset S6). Furthermore, we performed qPCR of *PaACS9*, *PaACS11*, *PaACO1*, *PaACO4*, *PaETR2*, and a senescence-related transcription factor gene, *PaWRKY23* (*Peaxi162Scf00770g00114*) (Ruan *et al.*, 2019). The results of qPCR assays (Supplementary Figs S9 and S10) for these genes were in agreement with the DEG results.

### *PaACLB1-B2* silencing-induced changes in the proteome profile of petunia corollas

We investigated the proteome in petunia corollas treated with pTRV2-PaACLB and pTRV2. Three biological replicates were analysed for each treatment. In total, 6200 protein groups were identified from petunia, among which 5343 proteins were quantified (Supplementary Dataset S8). A total of 345 proteins were up-regulated and 182 proteins were down-regulated (with a threshold of 1.2-fold) in *PaACLB1-B2*-silenced plants compared with the control (*P*<0.05), with a high degree of repeatability (Supplementary Dataset S9; Supplementary Fig. S11).

**Fig. 7.**
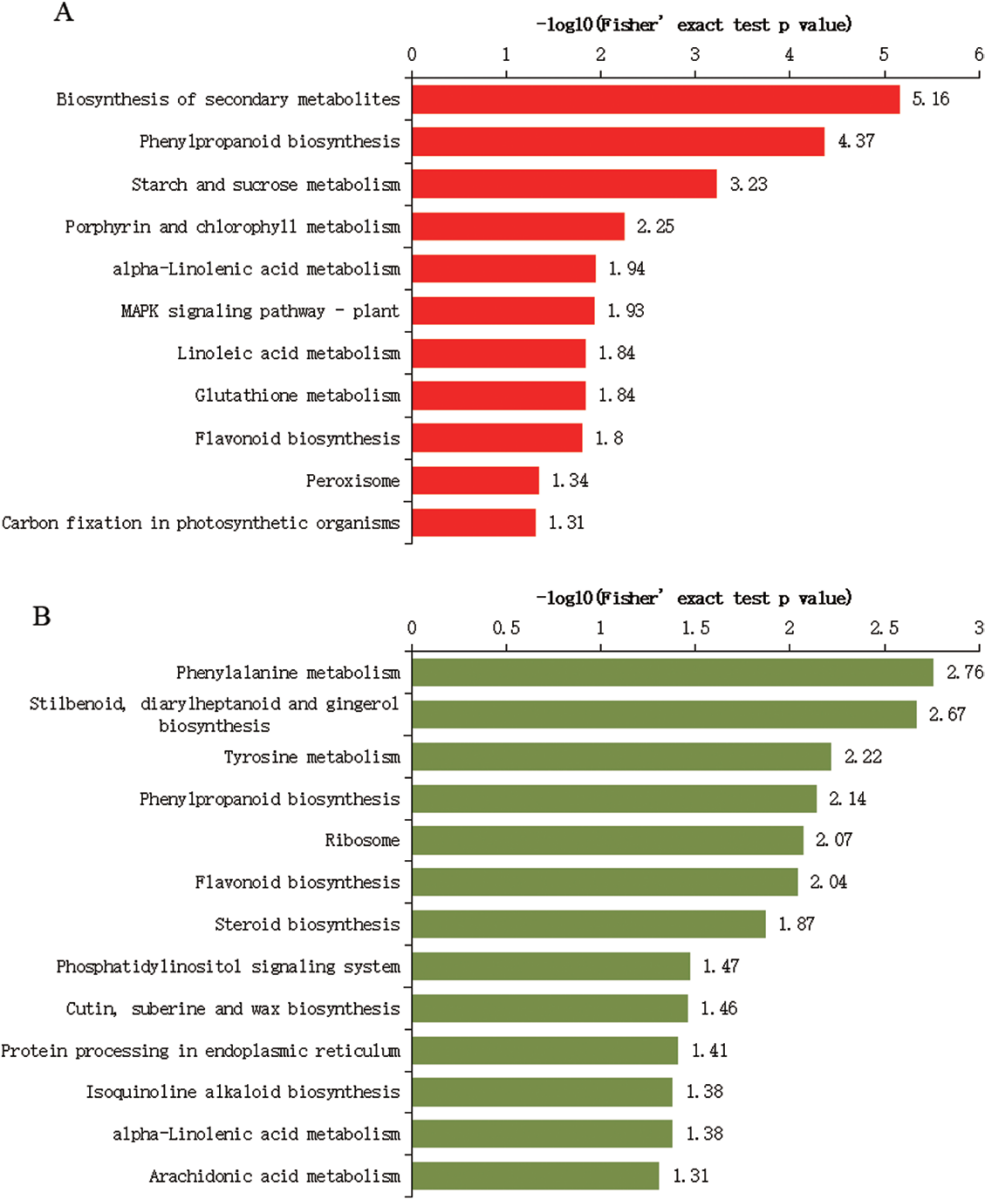
KEGG pathway enrichment analysis of differentially expressed proteins in *PaACLB1-B2*-silenced petunia corollas compared with controls. (A) Up-regulated proteins; (B) down-regulated proteins. The significance level was set at *P*<0.05 (Fisher’s exact test). Data are from Supplementary Dataset S10.

The up- and down-regulated proteins were enriched in 11 and 13 KEGG metabolic pathways, respectively (Supplementary Dataset S10). Many of the up-regulated proteins were enriched in the biosynthesis of secondary metabolites, phenylpropanoid biosynthesis, starch and sucrose metabolism, porphyrin and chlorophyll metabolism, alpha-linolenic acid metabolism, MAPK signalling pathway, linoleic acid metabolism, glutathione metabolism, flavonoid biosynthesis, peroxisome, and carbon fixation in photosynthetic organisms (Fig. 7A). Many of the down-regulated proteins were enriched in phenylalanine metabolism, stilbenoid, diarylheptanoid and gingerol biosynthesis, tyrosine metabolism, phenylpropanoid biosynthesis, ribosome, flavonoid biosynthesis, steroid biosynthesis, phosphatidylinositol signalling system, cutin, suberine and wax biosynthesis, and protein processing in endoplasmic reticulum (Fig. 7B). These results further showed that acetyl-CoA is widely involved in secondary metabolism, including flavonoid biosynthesis and the stress response.

As expected, both PaACLB1 (3.0-fold) and PaACLB2 (>4.0-fold) were significantly down-regulated. Unexpectedly, the abundance of both PaACLA1 (1.4-fold) and PaACLA2 (6.2-fold) was significantly reduced. The abundance of PaACO1, PaCHSA (Peaxi162Scf00047g01225, 2.0-fold), four acyl transferases (Peaxi162Scf00003g00487, 1.2-fold; Peaxi162Scf00047 g00128, 1.2-fold; Peaxi162Scf00160g01037.1, 1.2-fold; Peaxi162Scf00075g01225, 1.3-fold) and two histone proteins (H2A.11, Peaxi162Scf71358g00001, and another histone protein, Peaxi162Scf00836g00036) was significantly increased (Supplementary Dataset S9). The abundance of all six differentially expressed proteins enriched in porphyrin and chlorophyll metabolism were up-regulated in *PaACLB1-B2*-silenced plants compared with controls (Supplementary Dataset S9).

**Fig. 8.**
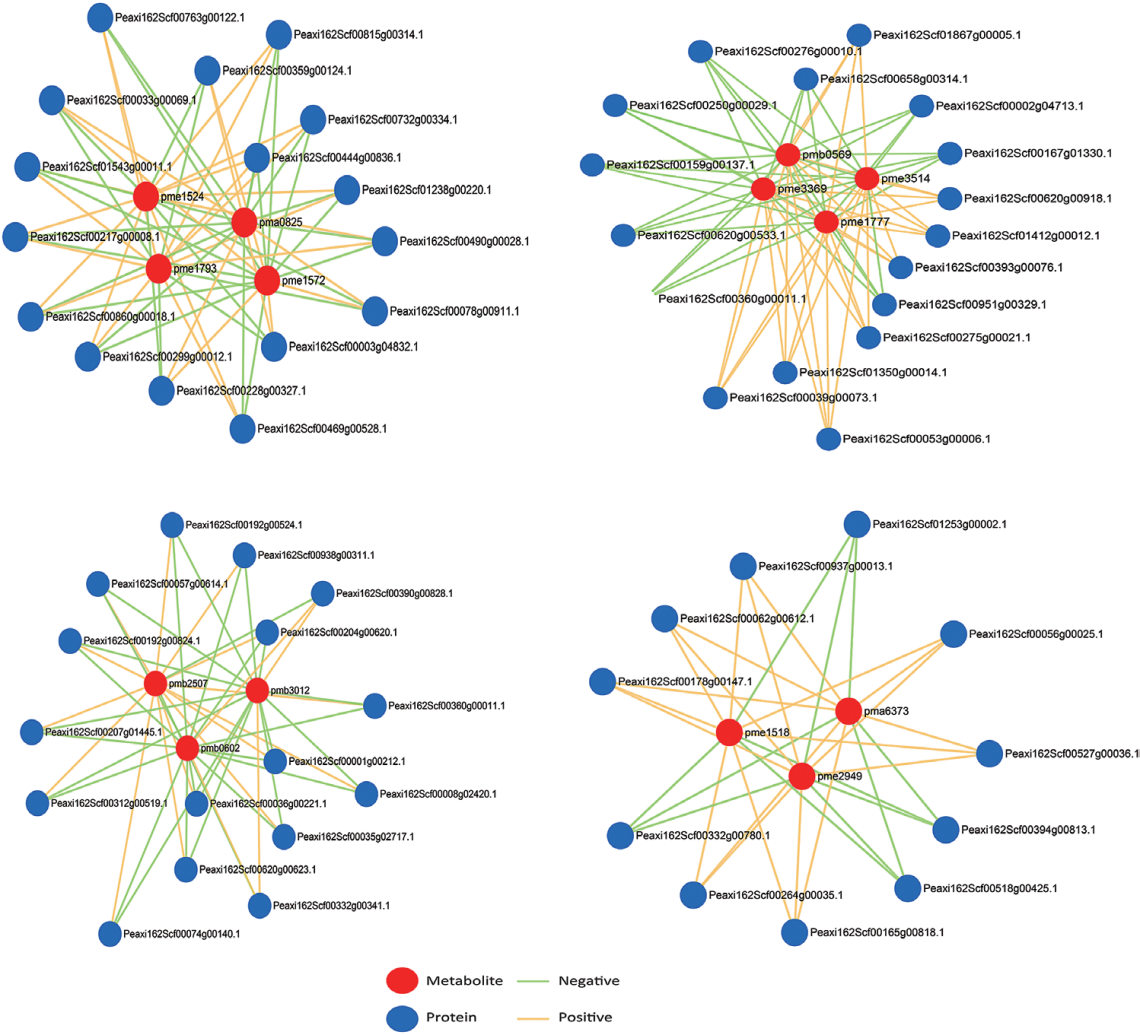
Metabolite–protein co-expression network of the significant differential flavonoid metabolites and differential proteins in the corollas of *PaACLB1-B2* silenced petunia plants and controls. The node size correlates with the ratio between responders and non-responders.

To study the potential relationship between differential proteins and differential metabolites, we analysed the KEGG pathway annotation of differential proteins and differential metabolites. Twenty pathways with both differential proteins and differential metabolites, which were enriched in the flavonoid biosynthesis pathway, were screened out (Supplementary Figs S12 and S13). The metabolite–protein co-expression network of 14 differential flavonoid metabolites and 58 differential proteins in the corollas of *PaACLB1-B2* silenced plants and controls is shown in Fig. 8.

### Effects of *PaACLB1-B2* silencing on lysine acetylation

Since acetyl-CoA is the substrate of lysine acetylation, the acetylated proteins and their modification sites were identified using TMT labelling and lysine acetylated (Kac) affinity enrichment followed by high-resolution LC-MS/MS in petunia corollas treated with pTRV2-PaACLB1-B2 or pTRV2. A total of 2210 lysine acetylation sites in 1148 protein groups were identified, among which 1744 sites in 921 proteins were accurately quantified (Supplementary Dataset S11). In the identification, the mass errors were lower than 10 ppm, confirming that the sample preparation reached the standard (Supplementary Fig. S14A). The length of most peptides was between 8 and 20 amino acids, consistent with the properties of tryptic peptides (Supplementary Fig. S14B). We subsequently used the quantification results of the global proteome to normalize the acetylome quantification data. Unexpectedly, 68 sites in 54 lysine acetylation proteins were quantified as up-regulated targets, and 40 sites in 38 lysine acetylation proteins were quantified as down-regulated targets at a threshold of 1.2 (*P*<0.05) in *PaACLB1-B2*-silenced plants compared with the control, with a high degree of repeatability (Supplementary Dataset S12; Supplementary Fig. S15). The acetylated proteins contained from one to 21 acetylation sites, and there were 678 acetylated proteins containing only one acetylation site, accounting for 59.0% of the total acetylated proteins. The proportions of proteins with two, three, four, or more modification sites were 19.2, 10.0, 5.1, and 6.6%, respectively. Several MS/MS spectra corresponding to sites from proteins that undergo acetylation are presented in Supplementary Fig. S16. These results are expected to supply valuable resources for post-translational modification studies in the future.

We performed enrichment pathway analysis based on the KEGG database using FoldChange (*P*<0.05). The proteins with up-regulated acetylation levels were enriched in one KEGG metabolic pathway, tyrosine metabolism (Supplementary Dataset S13; Supplementary Fig. S17A). The proteins with down-regulated acetylation levels were enriched in five KEGG metabolic pathways: oxidative phosphorylation, glycolysis/gluconeogenesis, pentose phosphate pathway, fructose and mannose metabolism, and purine metabolism (Supplementary Dataset S13; Supplementary Fig. S17B). These results further showed that the reduction of acetyl-CoA is associated with primary metabolism.

We further performed GO analysis of the proteins with differential acetylation levels (Supplementary Dataset S14). With regard to molecular function, many proteins with up-regulated acetylation levels were enriched in phosphoglycerate kinase activity, transferase activity, transferring acyl groups, acyl groups converted into alkyl on transfer, protein dimerization activity, carbon–nitrogen lyase activity, phosphotransferase activity, and carboxyl group as acceptor. Many proteins with down-regulated acetylation levels were enriched in fructose-bisphosphate aldolase activity, aldehyde-lyase activity, carbon–carbon lyase activity, lipid binding, lyase activity, nucleobase-containing compound kinase activity, metal cluster binding, and iron–sulfur cluster binding. These results showed that acetyl-CoA is involved in many post-translational modifications of proteins or transferring acyl or carboxyl groups of metabolites, and affects enzyme activities.

In terms of biological processes, a large proportion of proteins with up-regulated acetylation levels were enriched in nucleosome organization, DNA packaging, nucleosome assembly, chromatin assembly, protein–DNA complex subunit organization, chromatin assembly or disassembly, protein–DNA complex assembly, cellular component assembly, macromolecular complex assembly, and DNA conformation change. Many proteins with down-regulated acetylation levels were enriched in purine nucleoside metabolic process, purine ribonucleoside metabolic process, ribonucleoside metabolic process, nucleobase-containing small molecule metabolic process, purine-containing compound metabolic process, purine ribonucleoside triphosphate metabolic process, purine nucleoside triphosphate metabolic process, nucleoside metabolic process, glycosyl compound metabolic process, and ribonucleoside triphosphate metabolic process. These results showed that the reduction of acetyl-CoA resulted in the occurrence of a large number of nuclear events, including transcription regulation and DNA synthesis.

Unexpectedly, the levels of acetylation were down-regulated in 15 and up-regulated in 17 proteins located in the cytoplasm, and the levels of acetylation of many proteins located in the chloroplast, nucleus, and plasma membrane were significantly changed because acetyl-CoA usually does not penetrate cell membranes. These results indicated that acetyl-CoA deficiency, as caused by reduced PaACL activity, affects crosstalk between different intracellular organelles and compartments, which is a response to metabolic stress and causes significant metabolic adaptation.

**Fig. 9.**
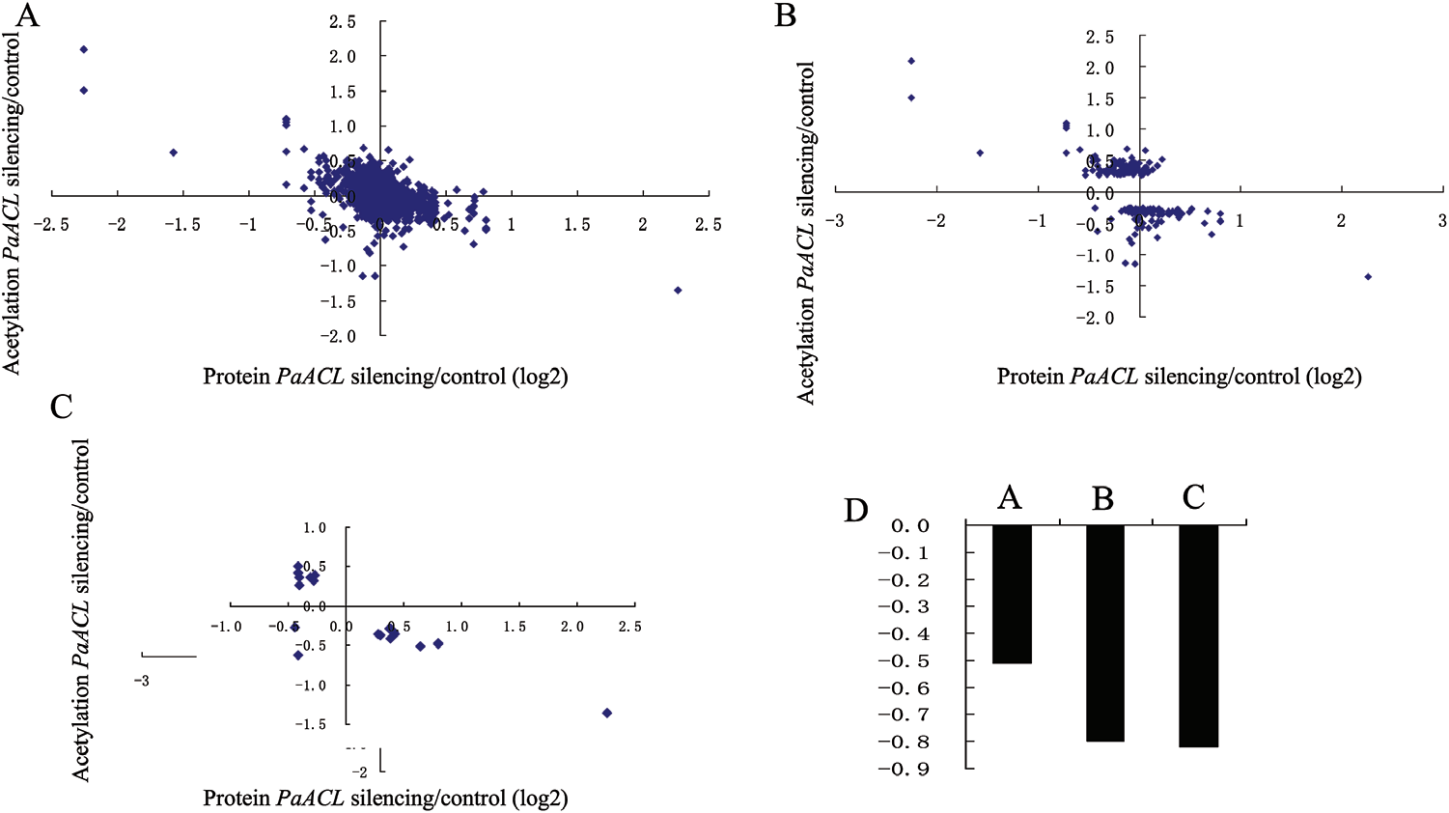
Concordance between changes in proteins and their acetylation. (A–C) Correlation between protein and acetylation fold changes in petunia corollas treated with pTRV2-PaACLB1-B2 compared with those treated with pTRV2 (control) for all acetylation/protein pairs (A), significantly changed proteins (B), and significantly changed acetylation sites (C). (D) Pearson correlations of the comparisons shown in (A–C).

The acetylation of PaACLA1 (six sites), PaACLB1 (one site), and PaACLB2 (two sites) was detected, and interestingly, the differential acetylation levels of two, one, and two sites in PaACLA1, PaACLB1, and PaACLB2, respectively, were all up-regulated in PaACLB1-B2-silenced plants compared with the control. Thus, a negatively regulated loop was formed, such that PaACL negatively regulated the acetylation level of PaACL itself by regulating the level of acetyl-CoA (Fig. 9). Whether the acetylation of PaACL enhances enzyme activity requires further study. The acetylation levels of eight sites of two histones, including histone H2A.11, were up-regulated (Supplementary Dataset S12).

In addition, we identified a total of 12 conserved motifs of acetylated sites (Supplementary Fig. S18A) and analysed the frequencies of neighbouring amino acid residues for acetylated lysines using iceLogo (Supplementary Fig. S18B) (Colaert *et al.*, 2009). To study the evolutionary conservation of acetylated lysine and non-acetylated lysine in plants, we aligned petunia proteins with their respective orthologues from three other species. The results unexpectedly showed that acetylated lysines are significantly more conserved than non-acetylated lysines, suggesting that acetylated lysines maintain a stronger selective pressure than non-acetylated lysines in plants (Supplementary Fig. S19).

We selected three proteins, PaACO1 (Peaxi162Scf00047g01927), PaCHSA, and PamCS (mitochondrial citrate synthase, Peaxi162Scf00402g00612), and one additional protein, Argonaute (PaARG717Kac, Peaxi162Scf00050g00169), to examine their protein and acetylation abundance by western blotting. The results of western blotting using antibodies raised against these proteins were consistent with the results of the proteome and acetylome analyses (Supplementary Fig. S20).

### Correlation between the global proteome and acetylome

Our data on the proteome and acetylome of pTRV2- and pTRV2-PaACLB1-B2-treated corollas allowed us to examine the correlation between the global proteome and acetylome. There were 1744 quantified Kac sites in 920 quantified proteins in this study (Supplementary Dataset S15). Of the 920 quantified proteins, 25 proteins were down-regulated and 58 were up-regulated. Pearson’s correlation coefficient was calculated as –0.51 when all quantified proteins were considered in terms of their quantified acetylation (Fig. 9A, D) and increased to –0.80 when all significantly altered proteins were considered in terms of their quantified acetylation (Fig. 9B, D). For acetylation/protein pairs with significantly altered proteins and acetylation, a larger negative correlation was observed (*r*=–0.82; Fig. 9C, D). Therefore, the global proteome and acetylome were negatively correlated.

Interestingly, in acetylation/protein pairs with significantly altered proteins and acetylation, the changed direction of all acetylation sites in one protein was the same, which allowed us to perform KEGG pathway analysis of the differential proteins with acetylation sites in the opposite direction. These proteins were enriched in two KEGG pathways, fatty acid degradation and alpha-linolenic acid metabolism (Supplementary Dataset S16, Supplementary Fig. S21).

## Discussion

Acetyl-CoA is a central metabolite and the acetyl source for acetylation of many metabolites and proteins (Oliver *et al.*, 2009; Chen *et al.*, 2017a). In this study, we investigated the effects of the reduction of ACL-derived cytosolic acetyl-CoA on growth, development, and the global metabolome, and performed transcriptome, proteome, and acetylome analyses of petunia corollas.

In this study, we first analysed the spatial and temporal expression of four genes encoding two subunits of PaACL during plant growth and development. The mRNAs of four *PaACLs* were detected in all organs (roots, stems, leaves, and corollas) examined, but were present at different levels in different organs and stages of development (Fig. 1Supplementary Fig. S2), indicating the importance of PaACL in the growth and development of plants. Although the expression patterns of the four genes were not completely consistent, there was functional redundancy in the different genes encoding each subunit.

We produced VIGS-mediated *PaACLA*- and *PaACLB*-silenced plants. Silencing of both *PaACLB1* and *PaACLB2* (*PaACLB1-B2*) or both *PaACLA1* and *PaACLA2* (*PaACLA1-A2*) resulted in similar abnormal growth and development of plants, while silencing of any single *PaACL* gene did not result in any visible phenotype change; this demonstrated that both ACL subunits were required to form a functional ATP-citrate lyase, and that there was functional redundancy between *PaACLB1* and *PaACLB2*, and between *PaACLA1* and *PaACLA2*. Similarly, both acl1 and acl2 subunits are required to form a functional ATP-citrate lyase in *Aspergillus niger* (Chen *et al.*, 2013).

Silencing of *PaACLB1-B2* reduced the content of acetyl-CoA and resulted in abnormal leaf and flower development (Fig. 2D–JSupplementary Fig. S3E). Small flower sizes, necrotic tissues in the corolla edge, and short filaments, styles, and pedicels were observed in *PaACLB1-B2*-silenced plants. Consistently, the adaxial and abaxial epidermal cells of corollas in the *PaACLB1-B2*-silenced plants were small compared with the control, as observed by scanning electron microscopy (Fig. 4). In *A. thaliana*, plants with reduced ACL activity showed a similar phenotype (Fatland *et al.*, 2005). In addition, *PaACLB1-B2* silencing reduced the fertility of both stamens and pistils, accelerated flower senescence, and increased ethylene production, features that were not reported in *A. thaliana.* These results indicate the important roles of *PaACL* in plant growth and development.

Consistent with *A. thaliana* with antisense *ACLA* (Fatland *et al.*, 2005), *PaACLB1-B2* silencing changed the chlorophyll content and anthocyanin accumulation in both corollas and seeds (Fig. 3A, C; Supplementary Fig. S3J). In *PaACLB1-B2*-silenced plants, the amino acid content in leaves changed greatly, especially the accumulation of proline (Supplementary Dataset S2). Proline accumulation has been reported during conditions of drought, oxidative stress, and in response to biotic stresses (Fabro *et al.*, 2004; Choudhary *et al.*, 2005; Haudecoeur *et al.*, 2009; Yang *et al.*, 2009). Fatland *et al.* (2005) suggested that the shortage of acetyl-CoA in *A. thaliana* resulted in physiological stress, termed ‘metabolic stress’. In this study, the shortage of acetyl-CoA induced by *PaACLB1-B2* silencing resulted in abnormal leaf and flower development, necrotic tissues in the corolla edge, acceleration of flower senescence, and higher proline content, which further supports the hypothesis that metabolic stress is induced by *ACL* silencing.

Metabolome analysis of petunia corollas showed that *PaACLB1-B2* silencing changed the metabolome profile (Supplementary Dataset S2). The accumulation of primary metabolites (e.g. soluble sugars, amino acids, organic acids, lipids) exerts resistance to metabolic stress and maintains the metabolism of cells. Secondary metabolites may play an important role as antioxidants; these include flavonoids, phenolamides, and coumarins, and other metabolites that were significantly induced or inhibited by metabolic stress in *PaACLB1-B2*-silenced plants. The involvement of phenolamides in plant defence against biotic and abiotic stresses has also been proposed (Buanafina, 2009; Park *et al.*, 2009; Bassard *et al.*, 2010; Demkura *et al.*, 2010; Kaur *et al.*, 2010; Onkokesung *et al.*, 2012). Some coumarin compounds have been identified as phytotoxic metabolites and function in the defence response of plants against biotic and abiotic stresses (Baillieul *et al.*, 2003; Shimizu *et al.*, 2005; Prats *et al.*, 2007; Gnonlonfin *et al.*, 2012; Sun *et al.*, 2014; Liu *et al.*, 2017b; Duan *et al.*, 2019). Although acetyl-CoA is the precursor of many metabolites, including flavonoids, *PaACLB1-B2* silencing up-regulated the content of 66 metabolites, including 29 downstream metabolites of acetyl-CoA metabolism, while only 30 metabolites were down-regulated. In *A. thaliana* plants in which ACLA was suppressed, the concentration of anthocyanins, a group of flavonoids, in rosettes and inflorescence stems increased. These results demonstrated the reconstitution of metabolic homeostasis in the case of a shortage of acetyl-CoA in *PaACLB1-B2*-silenced petunia corollas.

The transcriptome analysis in this study showed that *PaACLB1-B2* silencing activated the transcription of many genes, especially genes involved in the biosynthesis of secondary metabolites, phenylpropanoid biosynthesis, and arginine and proline metabolism (Supplementary Dataset S4). In line with these results, in the metabolome analysis, the contents of many secondary metabolites, including phenylpropanoids and proline, increased. These results showed that *PaACLB1-B2* silencing changed the transcriptome profile to some extent to maintain metabolic homeostasis.

The proteome analysis showed that 345 proteins were up-regulated and only 182 proteins were down-regulated in *PaACLB-B2*-silenced plants compared with the control. Most differentially expressed proteins were enriched in KEGG pathways including biosynthesis of secondary metabolites, phenylpropanoid biosynthesis pathways, and phenylalanine metabolism. In the proteome and metabolome analyses, both the differential proteins and the differential metabolites were enriched in the flavonoid biosynthesis pathway. In addition, many up-regulated proteins were enriched in porphyrin and chlorophyll metabolism and starch and sucrose metabolism, and the contents of chlorophyll and all differential carbohydrate metabolites were up-regulated. Moreover, there were 20 pathways in which both differential proteins and differential metabolites were distributed, some of which were enriched in the flavonoid biosynthesis pathway. These results show that *PaACLB1-B2* silencing changed the proteome profile to maintain metabolic homeostasis. The down-regulated proteins were enriched in the KEGG pathways stilbenoid, diarylheptanoid and gingerol biosynthesis, tyrosine metabolism, flavonoid biosynthesis, and cutin, suberine and wax biosynthesis, most likely indicating the feedback regulation of metabolites on the abundance of synthetic enzymes.

Analysis of qPCR and transcriptome data showed that the expression of *PaACS9*, *PaACS11*, *PaACO1*, and *PaACO4*, which encode the key enzymes in ethylene biosynthesis, was up-regulated, and proteome data showed that *PaACO1* abundance was up-regulated compared with the control. A recent study in rice (*Oryza sativa*) showed that a reduction in OsACL-A2 abundance resulted in evident phenotypes, with small lesion-mimic leaves and enhanced immunity to bacterial blight, and an increase in the expression of senescence-related genes (Ruan *et al.*, 2019). It is possible that the metabolic stress induced by the reduction of acetyl-CoA in *PaACLB1-B2*-silenced plants promoted flower senescence by increasing the expression of cell death- and senescence-related genes, including *PaACO* and *PaACS* (Supplementary Fig. S22). These results indicate that the changes in transcriptome and proteome profiles are associated with abnormal growth and development caused by *PaACLB1-B2* silencing.

Acetyl-CoA is the source of acetyl for protein acetylation, including histone acetylation, which promotes gene expression. Unexpectedly, protein lysine acetylome analysis showed that the reduction of acetyl-CoA up-regulated the acetylation level of more proteins of different localization. Consistently, the abundance of four acyl-transferases was up-regulated (Supplementary Dataset S6). Only a minority of differentially Lys-acetylated proteins (~30%) was localized in the cytosol of ACL-depleted plants. Since other compartments have their own acetyl-CoA sources, the major fraction of affected proteins appears to be perturbed due to pleiotropic effects. This may also explain why more proteins were more frequently Lys-acetylated in the mutants. The proteins with different acetylation levels were enriched in several KEGG metabolic pathways associated with primary metabolism (Supplementary Dataset S13). The results of GO analysis showed that the proteins with different levels of acetylation were enriched in many post-translational modifications of proteins or transferring acyl or carboxyl groups of metabolites, and affected the enzyme activities. It is possible that acetyl-CoA may be used effectively under conditions of a shortage of acetyl-CoA. These results showed that *PaACLB1-B2* silencing changed the acetylome profile to maintain metabolic homeostasis.

Two categories of enzymes, CHS and CHI, play critical roles in flavonoid synthesis. Interestingly, CHS and CHI usually play key roles in the plant response to stress (Koes *et al.*, 2005; Falcone Ferreyra *et al.*, 2012; Ishida *et al.*, 2016). *AtCHS* expression was significantly increased concomitantly with the accumulation of anthocyanins in *A. thaliana* plants subjected to salt stress (Piao *et al.*, 2001). In this study, the abundance of PaCHSA and the acetylation level of PaCHI increased, which explained, at least partially, the cause of the increase in many flavonoids in the corollas of *PaACLB1-B2* silenced plants. Correspondingly, the increase in the abundance of PaANS could be connected with the increase in some anthocyanin metabolites (Supplementary Fig. S23). The abundance of the biosynthetic enzyme of proline, PaP5CS1 (Peaxi162Scf00658g00314), whose transcript level did not change, was significantly up-regulated, and could be connected to the observed increase of proline contents. These results further support that metabolic homeostasis is maintained in *PaACLB1-B2* silenced plants by changing the proteome and acetylome.

In mammalian cells, ACL is required for increases in histone acetylation in response to growth factor stimulation and during differentiation, and fluctuation of cellular acetyl-CoA levels affects histone acetylation in yeast and mammalian cells at multiple lysine residues of histone (H3 and H4) tails (Wellen *et al.*, 2009). In *A. thaliana*, the increase of H3K27 acetylation (H3K27ac) is dependent on cytoplasmic ACL (Chen *et al.*, 2017a). In this study, we did not detect a change in the acetylation level of H3K27, but we did detect up-regulation of the levels of acetylation of histones H2A.11 and another histone superfamily protein. Obviously, these were not direct consequences of the reduction of acetyl-CoA, which could be attributed to *PaACLB1-B2* silencing-induced metabolic stress.

Silencing of *PaACLA1-A2* or *PaACLB1-B2* did not result in a change in the mRNA levels of the *PaACLB*s and *PaACLA*s (Supplementary Figs S4 and S5), respectively, but resulted in not only a significant reduction in PaACLB1 and PaACLB2 abundance (as expected) but also a significant reduction in PaACLA1 and PaACLA2 abundance. Similarly, in *A. thaliana*, the abundance of both ACLA and ACLB is reduced in antisense *ACLA* plants (Fatland *et al.*, 2005). There is no sequence similarity between any of the *ACLA* and *ACLB* genes; thus, this coordination in expression is not a co-suppression mechanism. We speculate that coordination between ACLA and ACLB expression occurs via a post-transcriptional mechanism and that excess ACLA subunits may be turned over in the absence of ACLB. In addition, the levels of acetylation of PaACLA1, PaACLB1, and PaACLB2 were up-regulated in *PaACLB1-B2*-silenced plants, and we could not rule out the possibility that increasing the acetylation of PaACLs increased their activities.

Previous studies suggested that the proteome and ubiquitylome were negatively correlated in petunia corollas and human cells, since ubiquitination plays important roles in protein degradation (Guo *et al.*, 2017; Jiang *et al.*, 2018). In this study, surprisingly, the global proteome and acetylome were negatively correlated in petunia corollas treated with pTRV2-PaACLB1-B2 and pTRV2 (Supplementary Dataset S15), implying that proteome expression levels were negatively regulated by acetylation in *PaACLB1-B2*-silenced plants compared with the control. It is possible that under conditions of acetyl-CoA deficiency, the abundance of some proteins with high acetylation levels was reduced to save the acetyl-CoA source. In eukaryotes, acetylation has been shown to affect the rate of turnover of certain proteins (Hershko *et al.*, 1984). The negative correlation between the proteome and acetylome might occur only under particular conditions, such as acetyl-CoA deficiency.

**Fig. 10.**
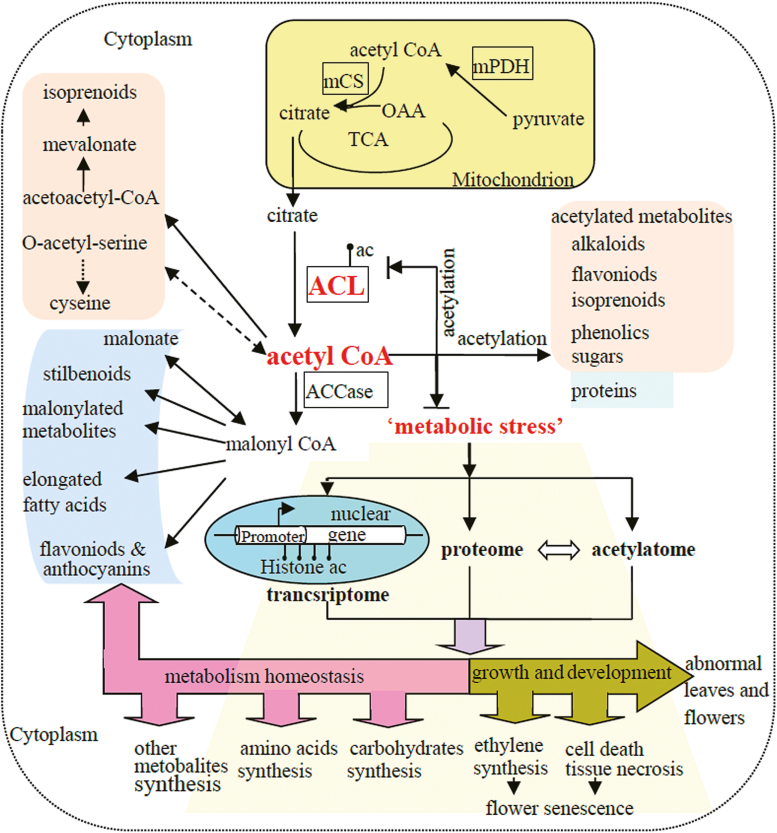
Acetyl-CoA metabolism and the regulation of *PaACLB1-B2* silencing-induced metabolic stress in petunia. ACL, ATP-citrate lyase; ac, acetylation; ACCase, acetyl-CoA carboxylase; mCS, mitochondrial citrate synthase; OAA, oxaloacetate; TCA, tricarboxylic acid cycle; mPDH, mitochondrial pyruvate dehydrogenase.

In conclusion, our results show that petunia PaACL plays important roles in the synthesis of most secondary metabolites and in plant growth and development, including flower senescence. Moreover, the reduction in cytosolic acetyl-CoA increased the content of many secondary metabolites and produced a new metabolic homeostasis by metabolic stress-induced changes in the transcriptome, proteome, and acetylome profiles of petunia corollas (Fig. 10). In addition, the negative correlation between the proteome and acetylome, which has not been reported previously, might occur only under specific conditions, such as metabolic stress induced by *PaACL* silencing.

## Supplementary data

Supplementary data are available at *JXB* online.

Table S1. Specific primer sequences used for full-length *ACL* gene isolation from *Petunia hybrida* ‘Ultra’.

Table S2. Primer sequences used in quantitative real-time PCR.

Table S3. Specific primer sequences used for VIGS vector construction.

Table S4. Peptides expressed in *Escherichia coli* of three proteins, PamCS, PaPDC2, and PaGELP.

Table S5. Effects of *PaACLB1-B2* silencing on the plants and cell size.

Fig. S1. Predicted amino acid sequence alignments and neighbour-joining trees of ACLAs and ACLBs.

Fig. S2. Expression patterns of *PaACLA*s and *PaACLB*s determined by quantitative real-time PCR using *Actin* as the internal reference gene.

Fig. S3. Phenotypic alteration of VIGS-mediated silencing of *PaACLB1* and *PaACLB2* in plants.

Fig. S4. Expression of *PaACLA*s and *PaACLB*s determined using quantitative real-time PCR.

Fig. S5. Expression of *PaACLA*s and *PaACLB*s determined by quantitative real-time PCR using *Actin* as the internal reference gene.

Fig. S6. ACL activities of *PaACLB1-B2* silenced flowers and controls.

Fig. S7. Repeatability analysis of metabolome and transcriptome.

Fig. S8. KEGG pathway enrichment analysis of differentially expressed genes in petunia corollas of *PaACLB1-B2*-silenced plants and controls.

Fig. S9. Confirmation of expression data for six senescence-related genes by quantitative real-time PCR using *Cyclophilin* as the internal reference gene.

Fig. S10. Confirmation of expression data for six senescence-related genes by quantitative real-time PCR using *Actin* as the internal reference gene.

Fig. S11. Repeatability test between samples in the proteome of petunia corollas of *PaACLB1-B2*-silenced plants and controls.

Fig. S12. Distribution of KEGG pathways of differential proteins and differential metabolites.

Fig. S13. Bubble chart of KEGG enrichment analysis.

Fig. S14. Quality control of mass spectrometry data.

Fig. S15. Repeatability test between samples in protein lysine acetylome.

Fig. S16. MS/MS spectra of lysine acetylation of several proteins.

Fig. S17. KEGG pathway enrichment analysis of proteins with up-regulated and down-regulated Kac sites.

Fig. S18. Motif analysis of all the identified Kac sites in petunia.

Fig. S19. Evolutionary conservation of acetylated and non-acetylated lysines on protein orthologues in selected species.

Fig. S20. Confirmation of proteome and acetylome data.

Fig. S21. Differential proteins with acetylation sites in the opposite direction were enriched in the fatty acid degradation and alpha-linolenic acid metabolism KEGG pathways.

Fig. S22. Effects of *PaACLB1-B2* silencing on the proteins engaged in ethylene biosynthesis in petunia.

Fig. S23. Effects of *PaACLB1-B2* silencing on anthocyanin and other flavonoid biosynthesis and the proteins engaged in their biosynthesis pathway in petunia.

Dataset S1. Total metabolites identified in petunia corollas.

Dataset S2. Differential metabolites in petunia corollas of *PaACLB1-B2* silenced plants and controls.

Dataset S3. KEGG pathway analysis of differential metabolites in petunia corollas of *PaACLB1-B2*-silenced plants and controls.

Dataset S4. Transcriptome analysis of corollas from the *PaACLB1-B2*-silenced plants and controls.

Dataset S5. Differential mRNAs in corollas from the *PaACLB1-B2*-silenced plants and controls.

Dataset S6. KEGG pathway enrichment of differential mRNAs in corollas from the *PaACLB1-B2*-silenced plants and controls.

Dataset S7. GO enrichment of differential mRNAs in corollas from the *PaACLB1-B2*-silenced plants and controls.

Dataset S8. Proteome analysis of corollas from the *PaACLB1-B2*-silenced plants and controls.

Dataset S9. Differential proteins in corollas from the *PaACLB1-B2*-silenced plants and controls.

Dataset S10. KEGG pathway enrichment of differential proteins in corollas from the *PaACLB1-B2*-silenced plants and controls.

Dataset S11. Acetylome analysis of the corollas from the *PaACLB1-B2*-silenced plants and controls.

Dataset S12. Differential acetylation sites in proteins in corollas from the *PaACLB1-B2*-silenced plants and controls.

Dataset S13. KEGG pathway enrichment of proteins with differential acetylation sites in corollas from the *PaACLB1-B2*-silenced plants and controls.

Dataset S14. GO enrichment of proteins with up- and down-regulated acetylation sites in corollas from the *PaACLB1-B2*-silenced plants and controls.

Dataset S15. Connection of proteome and acetylome

Dataset S16. Differential proteins with opposite direction differential acetylation sites.

eraa208_suppl_Supplementary_Figures_S1-S23

eraa208_suppl_Supplementary_Tables_S1-S5

eraa208_suppl_Supplementary_Datasets_S1-S4

eraa208_suppl_Supplementary_Dataset_S5-S8

eraa208_suppl_Supplementary_Datasets_S9-S12

eraa208_suppl_Supplementary_Datasets_S13-S16

## Data Availability

The mass spectrometry proteomics data have been deposited to the ProteomeXchange Consortium via the PRIDE (https://www.ebi.ac.uk/pride/; Perez-Riverol *et al*., 2019) partner repository with the dataset identifier PXD015480. The raw RNA sequence data have been submitted to NCBI with accession number PRJNA577189 (https://dataview.ncbi.nlm.nih.gov/object/PRJNA577189).
