# Supplementary material for: PaACL silencing accelerates flower senescence and changes the proteome to maintain metabolic homeostasis in Petunia hybrida
Source: J Exp Bot. 2020 May 4;71(16):4858–76. doi: 10.1093/jxb/eraa208 (PMC7475263; doi:10.1093/jxb/eraa208)
Supplement: eraa208_suppl_Supplementary_Figures_S1-S23 [file eraa208_suppl_supplementary_figures_s1-s23.pdf]

A

```
AtACLA1   MARKKIREYDSKRLVKEHFKRLSGKELPIRSVQINETTDLNELVEKEPWLSSEKLVVKPDMLFGKR   66
AtACLA2   MARKKIREYDSKRLVKEHFKRLSGQELPIRSVQINQETDLNELVEREPWLSSEKLVVKPDMLFGKR   66
AtACLA3   MARKKIREYDSKRLLKEHLKRLANIDLQIRSAQVTESTDFTELTNQESWLSSTKLVVKPDMLFGKR   66
SlACLA1   MARKKIREYDSKRLLKEHFKRLGGYDLAIKSAQVTESTDINELAEKEPWLNSTKLVVKPDMLFGKR   66
SlACLA2   MARKKIREYDSKRLLKEHFKRLAGYDLAIKSAQITESTDLNELVEKEPWVSSKKLAVKPDMLFGKR   66
SlACLA3   MARKKIREYDSKRLLREHLKRLAGIDLQICSAQVTESTDFTELTNKEPWLSSTKLVVKPDMLFGKR   66
PaACLA1   MARKKIREYDSKRLLKEHFKRLAGYELAIKSAQVTESTDFNELTEKEPWLSSTKLVVKPDMLFGKR   66
PaACLA2   MARKKIREYDSKRLLKEHLKRLAAIDLKICSAQVTESTDFTELTNKEPWLSSTKLVVKPDMLFGKR   66
Consensus markkireydskrl  eh krl    l i s q    td  el   e w  s kl vkpdmlfgkr

AtACLA1   GKSGLVALKLDFADVATFVKERLGKEVEMSGCKGPITTFIVEPFVPHNEEYYLNVVSDRLGCSISF   132
AtACLA2   GKSGLVALNLDFADVATFVKERLGKEVEMSGCKGPITTFIVEPFVPHNEEFYLNIVSDRLGCSISF   132
AtACLA3   GKSGLVALKLDLAEVADFVKARLGTEVEMGGCKAPITTFIVEPFVPHDQEYYLSIVSDRLGCTISF   132
SlACLA1   GKSGLVALNLDLAQVATFVKERLGKEVEMGGCKGPITTFIVEPFIPHNEEFYLNIVSDRLGCSVSF   132
SlACLA2   GKSGLVALNPDLAQVALLLKERLGKEVDMSGCKGPITTFIVEPFVPHNEEFYLNIVSDRLGCSVSF   132
SlACLA3   GKSGLVALNLDLAGVAEFVKTRLGVEVEMGGCKAPITTFIVEPFVPHDQEYYLSIVSERLGCTISF   132
PaACLA1   GKSGLVALNLDLAQVAAFVKERLGKEVEMGGCKGPITTFIVEPFVPHNEEYYLNIVSERLGCSISF   132
PaACLA2   GKSGLVALNLDLAAVAEFVKARLGVEVEMGGCKAPITTFIVEPFVPHDQEYYLSIVSERLGCTISF   132
Consensus gksglval  d a va   k rlg ev m gck pittfivepf ph  e yl  vs rlgc  sf

AtACLA1   SECGGIEIEENWDKVKTIFLPTGASLTPEICAPLVATLPLEIKAEIEEFIKVIFTLFQDLDFTFLE   198
AtACLA2   SECGGIDIEENWDKVKTITIPTGASLTSEICAPLVATLPLEIKGELEDFIQVIFTLFEDLDFTFLE   198
AtACLA3   SECGGIEIEENWDKVKTIFLPAEKSMTLEVCAPLIATLPLEVRAKIGNFIMGAFAVFQDLDFSFME   198
SlACLA1   SECGGIDIEENWDKVKTISVPTGTSFTSEICAPLVATLPLEIKGVIEEFLKVIYALFQDLDFTFLE   198
SlACLA2   SECGGIDIEENWDKVKTIFVPTGTSFTSEICAPLAATLPLEIKGVIEEFLKDVYTLFQDLDFTFLE   198
SlACLA3   SECGGIEIEENWDKVKTIFLPTEKPMTLEACAPLIATLPLEVRGKIGNFLMGVFDVFQDLDFSFIE   198
PaACLA1   SECGGIDIEENWDKVKTIFVPTGTSFTSEICAPLVATLPLEIKGVIEEFLKVVYALFQDLDFTFLE   198
PaACLA2   SECGGIEIEENWDKVKTIFLPTEKPMTLEACAPLIATLPLEVRGTIGNFLMGVFNVFQDLDFSFIE   198
Consensus secggi ieenwdkvkti  p     t e capl atlple       f       f dldf f e

AtACLA1   MNPFTLVDGSPYPLDMRGELDDTAAFKNFKKWGDIEFPLPFGRVMSPTESFIHGLDEKTSASLKFT   264
AtACLA2   MNPFTLVDGKPYPLDMRGELDDTAAFKNFKKWGDIEFPMPFGRVMSSTESFIHGLDEKTSASLKFT   264
AtACLA3   MNPFTLVDGEPFPLDMRGELDDTAAFKNFNKWGDIEFPLPFGRVLSSTENFIHGLDEKTSASLKFT   264
SlACLA1   MNPFALVEGKPYPLDMRGELDDTAAFKNFKKWGNIEFPLPFGRVMSATESFIHGLDEKTSASLKFT   264
SlACLA2   MNPFTLVEGKPYPLDMRGELDDTATFKNFKKWGNVEFPLPFGRVMSATESFIHGLDEKTSVSLKFT   264
SlACLA3   MNPFTLVNGEPYPLDMRGELDDTAAFKNFKKWGSIEFPLPFGRVLSPTESFIHSLDEKTSASLKFT   264
PaACLA1   MNPFTLVEGKPYPLDMRGELDDTAAFKNFKKWGNIEFPLPFGRVMSATESFIHGLDEKTSASLKFT   264
PaACLA2   MNPFTLVNGEPYPLDMRGELDDTAAFKNFKKWGSIEFPLPFGRVLSPTESFIHSLDEKTSASLKFT   264
Consensus mnpf lv g p pldmrgelddta fknf kwg  efp pfgrv s te fih ldekts slkft

AtACLA1   VLNPKGRIWTMVAGGGASVIYADTVGDLGYASELGNYAEYSGAPKEDEVLQYARVVIDCATANPDG   330
AtACLA2   VLNPKGRIWTMVAGGGASVIYADTVGDLGYASELGNYAEYSGAPKEDEVLQYARVVIDCATANPDG   330
AtACLA3   VLNPKGRIWTMVAGGGASVIYADTVGDLGYASELGNYAEYSGAPNEEEVLQYARVVIDCATTDPDG   330
SlACLA1   VLNPKGRIWTMVAGGGASVIYADTVGDLGYASELGNYAEYSGAPNEEEVLQYARVVIDCATANPDG   330
SlACLA2   VLNPKGRIWTMVGGGGASVIYADNVGDLGYASELGNYTEYSGAPNEEEVLQYARVVIDCATADPYG   330
SlACLA3   VLNPKGRIWTMVAGGGASVIYADTVGDLGYASELGNYAEYSGAPNEEEVLQYARVVLDCATANPDG   330
PaACLA1   VLNPKGRIWTMVAGGGASVIYADTVGDLGYASELGNYAEYSGAPNEEEVLQYARVVIDCATANPDG   330
PaACLA2   VLNPKGRIWTMVAGGGASVIYADTVGDLGYASELGNYAEYSGAPNEEEVLQYARVVLDCATANPDG   330
Consensus vlnpkgriwtmv gggasviyad vgdlgyaselgny eysgap e evlqyarvv dcat  p g

AtACLA1   KSRALVIGGGIANFTDVAATFNGIIRALKEKEAKLKAARMHIFVRRGGPNYQKGLAKMRALGDDIG   396
AtACLA2   KSRALVIGGGIANFTDVAATFNGIIRALKEKEAKLKAARMHIFVRRGGPNYQKGLAKMRSLGDEIG   396
AtACLA3   RKRALLIGGGIANFTDVAATFNGIIRALREKETRLKASRMH........IYVRRGGPNYQTGLARM   388
SlACLA1   RKRALVIGGGIANFTDVAATFSGIIRALKEKESKLKAARMH.........................   371
SlACLA2   RKRALVIGWGIANFTDVAATFSGIIRALKEKEFKLKAARMH.........................   371
SlACLA3   RKRALIVGGGIANFTDVAATFNGIIRALREKEAKLKAARMH........IYVRRGGPNYQTGLAKM   388
PaACLA1   RKRALVIGGGIANFTDVAATFSGIIRALKEKESKLKAARMH.........................   371
PaACLA2   RKRALIIGGGIANFTDVAATFNGIIRALREKEAKLKAARMH........IYVRRGGPNYQTGLAKM   388
Consensus   ral  g gianftdvaatf giiral eke  lka rmh

AtACLA1   VPIE.......................VYGPEATMTGICKEAIQYITAAA...   423
AtACLA2   VPIE.......................VYGPEATMTGICKEAIQYITAAA...   423
AtACLA3   RALGEELGVPLEVYGPEA..................TMTGICKRAIDCIMLPD   423
SlACLA1   ..IYVRRGGPNYQKGLAKMRSLGEEIGIPIEVYGPEETMTGICKQAIECISAAA   423
SlACLA2   ..IYVRRGGPNYQKGLAKIWSLGEEIGIPIEVYGPEETRTGICKQAIECITA..   421
SlACLA3   RALGEELGVPLEVYGPEA..................TMTGICKRAIDCIMSEA   423
PaACLA1   ..IY.........................VYGPEETMTGICKQAIECITAAA   396
PaACLA2   RALGEELGVPLEVYGPEA..................TMTGICKRAIDCIMSEA   423
Consensus                                                 i
```

B

```
AtACLB1    MATGQLFSRNTQALFYNYKQLPIQRMLDFDFLCGRETPSVAGIINPGSEGFQKLFFGQEEIAIPVH    66
AtACLB2    MATGQLFSRTTQALFYNYKQLPVQRMLDFDFLCGRETPSVAGIINPGSEGFQKLFFGQEEIAIPVH    66
SlACLB1    MATGQLFSKTTQALFYNYKQLPIQRMLDFDFLCGRETPSVAGIINPGSEGFQKLFFGQEEIAIPVH    66
SlACLB2    MATGQLFSKTTQALFYNYKQLPIQRMLDFDFLCGRETPSVAGIINPGSEGFQKLFFGQEEIAIPVH    66
PaACLB1    MATGQLFSKTTQALFYNYKQLPIQRMLDFDFLCGRETPSVAGIINPGSEGFQKLFFGQEEIAIPVH    66
PaACLB2    MATGQLFSKTTQALFYNYKQLPIQRMLDFDFLCGRETPSVAGIINPGSEGFQKLFFGQEEIAIPVH    66
Consensus  matgqlfs  tqalfynykqlp qrmldfdflcgretpsvagiinpgsegfqklffgqeeiaipvh

AtACLB1    AAIEAACAAHPTADVFINFASFRSAAASSMAALKQPTIKVVAIIAEGVPESDTKQLIAYARANNKV   132
AtACLB2    AAIEAACAAHPTADVFINFASFRSAAASSMAALKQPTIKVVAIIAEGVPESDTKQLIAYARANNKV   132
SlACLB1    STIEAACAAHPTADVFINFASFRSAAASSMSALKQPTIKVAAIIAEGVPESDAKELIAYAKANNKV   132
SlACLB2    STVEAACAAHPTADVFINFASFRSAAASSMSALKQPTIRVVAIIAEGVPESDTKQLIGFAKANNKV   132
PaACLB1    STIEAACAAHPTADVFINFASFRSAAASSMSALKQPTIKVAAIIAEGVPESDAKQLIAYAKANNKV   132
PaACLB2    STVEAACAAHPTADVFINFASFRSAAASSLSALKQPTIRVVAIIAEGVPESDTKQLIGYAKANNKV   132
Consensus     eaacaahptadvfinfasfrsaaass  alkqpti v aiiaegvpesd k li  a annkv

AtACLB1    IIGPATVGGVQAGAFKIGDTAGTIDNIIQCKLYRPGSVGFVSKSGGMSNEMYNTIARVTDGIYEGI   198
AtACLB2    VIGPATVGGIQAGAFKIGDTAGTIDNIIQCKLYRPGSVGFVSKSGGMSNEMYNTVARVTDGIYEGI   198
SlACLB1    VIGPATVGGIQAGAFKIGDTAGTIDNIIQCKLYRPGSVGFVSKSGGMSNELYNSIARVTDGIYEGI   198
SlACLB2    VIGPATVGGIQAGAFKIGDTAGTIDNIIQCKLYRPGSVGFVSKSGGMSNELYNTIARVTDGVYEGI   198
PaACLB1    VIGPATVGGIQAGAFKIGDTAGTIDNIIQCKLYRPGSVGFVSKSGGMSNELYNTIARVTDGIYEGI   198
PaACLB2    VIGPATVGGIQAGAFKIGDTAGTIDNIIQCKLYRPGSVGFVSKSGGMSNELYNTIARVTDGVYEGI   198
Consensus   igpatvgg qagafkigdtagtidniiqcklyrpgsvgfvsksggmsne yn  arvtdg yegi

AtACLB1    AIGGDVFPGSTLSDHILRFNNIPQIKMVVVLGELGGRDEYSLVEAMKQGKVTKPVVAWVSGTCARL   264
AtACLB2    AIGGDVFPGSTLSDHILRFNNIPQIKMVVVLGELGGRDEYSLVEALKEGKVNKPVVAWVSGTCARL   264
SlACLB1    AIGGDVFPGSTLSDHVLRFNNIPQVKMVVVLGELGGRDEYSLVEALKQGKINKPVVAWVSGTCATL   264
SlACLB2    AIGGDVFPGSTLSDHVLRFNNIPQVKMIVVLGELGGRDEYSLVEALKQGKINKPVVAWVSGTCATL   264
PaACLB1    AIGGDVFPGSTLSDHVLRFNNIPQVKMVVVLGELGGRDEYSLVEAMKQGKINKPVVAWVSGTCATL   264
PaACLB2    AIGGDVFPGSTLSDHVLRFNNIPQVKMIVVLGELGGRDEYSLVEALKQGKISKPVVAWVSGTCATL   264
Consensus  aiggdvfpgstlsdh lrfnnipq km vvlgelggrdeyslvea k gk  kpvvawvsgtca l

AtACLB1    FKSEVQFGHAGAKSGGEMESAQAKNQALQDAGATVPTSFEALEVAIKETFDKLVEEGKVSPIKEVT   330
AtACLB2    FKSEVQFGHAGAKSGGEMESAQAKNQALIDAGAIVPTSFEALESAIKETFEKLVEEGKVSPIKEVI   330
SlACLB1    FKSEVQFGHAGAKSGGEMESAQAKNQALRDAGAIVPTSYEAFEGAIKDAFEKLVEAGKTTPVKEIT   330
SlACLB2    FKSEVQFGHAGAKSGGEMESAQAKNQALRDAGATVPTSYEAFEGAIKEAFEKLVSEGKTTPVKEIT   330
PaACLB1    FKSEVQFGHAGAKSGGEMESAQGKNQALRDAGAVVPTSYEAFEGAIKDTFEKLVEAGKTTPVKEVT   330
PaACLB2    FKSEVQFGHAGAKSGGEMESAQAKNQALKDAGAVVPTSYEAFEGAIKETFEQLTSEGKTTPVKEIT   330
Consensus  fksevqfghagaksggemesaq knqal daga vpts ea e aik  f  l   gk  p ke

AtACLB1    PPQIPEDLSSAIKSGKVRAPTHIISTISDDRGEEPCYAGVPMSSIIEQGYGVGDVISLLWFKRSLP   396
AtACLB2    PPQIPEDLNSAIKSGKVRAPTHIISTISDDRGEEPCYAGVPMSSIIEQGYGVGDVISLLWFKRSLP   396
SlACLB1    PPQIPEDLSTAIKSGKVRAPTHIISTISDDRGEEPCYAGVPMSSIVEQGLGVGDVISLLWFKRSLP   396
SlACLB2    PPQIPEDLNTAIKSGKVRAPTHIISTISDDRGEEPCYAGVPMSSIVEQGLGVGDVISLLWFKRSLP   396
PaACLB1    PPQIPEDLSTAIKSGKVRAPTHIISTISDDRGEEPCYAGVPMSSIVEQGLGVGDVISLLWFKRSLP   396
PaACLB2    PPQIPEDLNTAIKSGKVRAPTHIISTISDDRGEEPCYAGVPMSSIVEQGLGVGDVISLLWFKRSLP   396
Consensus  ppqipedl  aiksgkvrapthiistisddrgeepcyagvpmssi eqg gvgdvisllwfkrslp

AtACLB1    RYCTKFIEICIMLCADHGPCVSGAHNTIVTARAGKDLVSSLVSGLLTIGPRFGGAIDDAARYFKDA   462
AtACLB2    RYCTKFIEICIMLCADHGPCVSGAHNTIVTARAGKDLVSSLVSGLLTIGPRFGGAIDDAARYFKDA   462
SlACLB1    RYCTRFIEICVMLCADHGPCVSGAHNSIVTARAGKDLVSCLVSGLLTIGPRFGGAIDDAARYFKDA   462
SlACLB2    RYCTRFIEICTLLCADHGPCVSGAHNSIVTARAGKDLVSCLVSGLLTIGPRFGGAVDDAARYFKDA   462
PaACLB1    RYCTRFIEICVMLCADHGPCVSGAHNSIVTARAGKDLVSCLVSGLLTIGPRFGGAIDDAARYFKDA   462
PaACLB2    RYCARFIEICVMLCADHGPCVSGAHNSIVTARAGKDLVSCLVSGLLTIGPRFGGAVDDAARYFKDA   462
Consensus  ryc  fieic  lcadhgpcvsgahn ivtaragkdlvs lvsglltigprfgga ddaaryfkda

AtACLB1    CDRNLTPYEFVEGMKKKGIRVPGIGHRIKSRDNRDKRVELLQKFARSNFPAVKYMEYAVQVETYTL   528
AtACLB2    CDRNLTPYEFVEGMKKKGIRVPGIGHRIKSRDNRDKRVELLQKFARSNFPSVKYMEYAVTVETYTL   528
SlACLB1    YDKGLTPYEFVESMKKKGIRVPGIGHRIKRGDNRDKRVELLQRYARENFPSVKYMEYAVQVETYTL   528
SlACLB2    YDRGLTPYEFVESMKKRGIRVPGIGHRIKRGDNRDKRVELLQLYARENFPSVKYMEYAVQVETYTL   528
PaACLB1    YDKNLTPYEFVESMKKKGIRVPGIGHRIKRGDNRDKRVELLQAYAREHFPSVNYMEYAVQVETYTL   528
PaACLB2    YDRGLTPYEFVESMKKKGIRVPGIGHRIKRGDNRDKRVELLQLYARENFPSVKYMEYAVQVETYTL   528
Consensus   d  ltpyefve mkk girvpgighrik  dnrdkrvellq  ar  fp v ymeyav vetytl

AtACLB1    SKANNLVLNVDGAIGSLFLDLLAGSGMFTKQEIDEIVQIGYLNGLFVLARSIGLIGHTFDQKRLKQ   594
AtACLB2    SKANNLVLNVDGAIGSLFLDLLAGSGMFTKQEIDEIVQIGYLNGLFVLARSIGLIGHTFDQKRLKQ   594
SlACLB1    SKANNLVLNVDGAIGSLFLDLLAGSGMFTKPEIDEIVGIGYLNGLFVLARSIGLIGHTFDQKRLKQ   594
SlACLB2    SKANNLVLNVDGAIGSLFLDLLAGSGMFTKPEIDEIVEIGYLNGLFVLARSIGLIGHTFDQKRLKQ   594
PaACLB1    SKANNLVLNVDGAIGSLFLDLLAGSRMFTKPEIDEIVEIGYLNGLFVLARSIGLIGHTFDQKRLKQ   594
PaACLB2    SKANNLVLNVDGAIGTLFLDLLAGSGMFTKPEIDEIVEIGYLNGLFVLARSIGLIGHTFDQKRLKQ   594
Consensus  skannlvlnvdgaig lfldllags mftk eideiv igylnglfvlarsiglightfdqkrlkq

AtACLB1    PLYRHPWEDVLYT                                                         607
AtACLB2    PLYRHPWEDVLYT                                                         607
SlACLB1    PLYRHPWEDVLYT                                                         607
SlACLB2    PLYRHPWEDVLYT                                                         607
PaACLB1    PLYRHPWEDVLYT                                                         607
PaACLB2    PLYRHPWEDVLYT                                                         607
Consensus  plyrhpwedvlyt
```

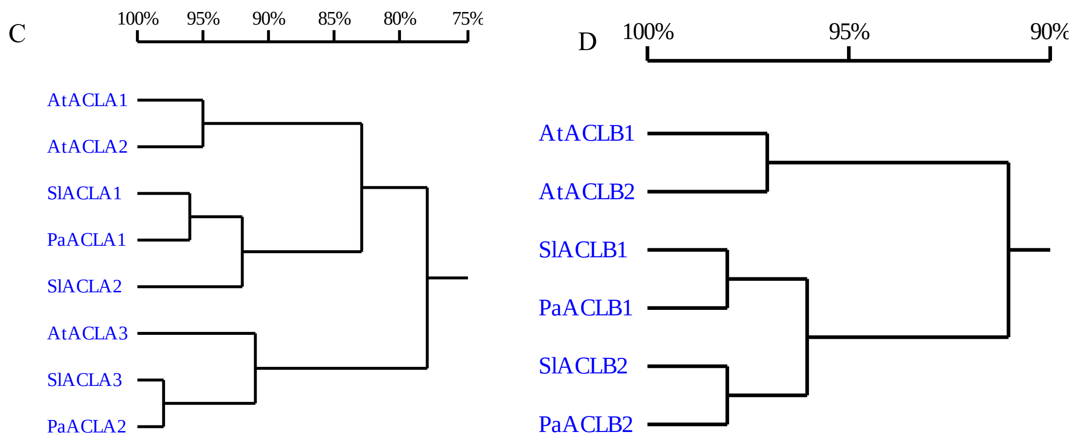

Figure S1 Predicted amino acid sequence alignments and neighbour-joining trees of ACLAs and ACLBs. (A) Predicted amino acid sequence alignments of petunia PaACLAs with *Arabidopsis thaliana* AtACLA1 (AT1G10670), AtACLA2 (AT1G60810), AtACLA3 (AT1G09430), *Solanum lycopersicum* SlACLA1 (Solyc05g005160.3), SlACLA2 (Solyc04g039670.3) and SlACLA3 (Solyc01g101040.3). Conserved residues are shaded in black. Grey shading indicates similar residues in six out of eight of the sequences. Slight grey shading indicates similar residues in five out of eight of the sequences. (B) Predicted amino acid sequence alignments of petunia PaACLBs with AtACLB1 (AT3G06650), AtACLB2 (AT5G49460), SlACLB1 (Solyc01g059880.3) and SlACLB2 (Solyc12g099260.1). Conserved residues are shaded in black. Grey shading indicates similar residues in five out of six of the sequences. Light grey shading indicates similar residues in four out of six of the sequences. (C) and (D) Neighbour-joining trees among proteins encoded by the *ACLA*- and *ACLB*-like genes using DNAMAN.

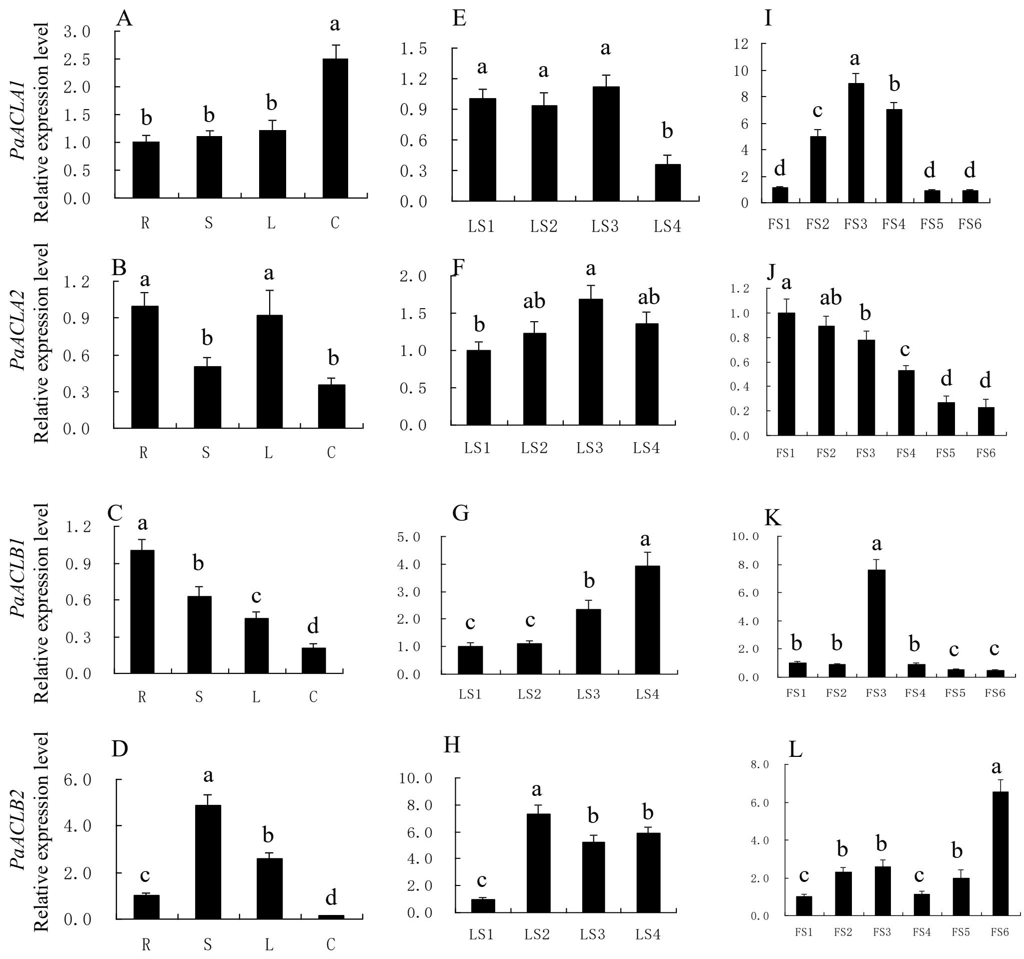

Figure S2 Expression patterns of *PaACLAs* and *PaACLBs* determined by quantitative real-time PCR using *Actin* as the internal reference gene. (A-D) Expression of *PaACLA1* (A), *PaACLA2* (B), *PaACLB1* (C) and *PaACLB2* (D) in different organs. R, roots; S, stems; L, leaves; C, corollas. (E-H) Expression of *PaACLA1* (E), *PaACLA2* (F), *PaACLB1* (G) and *PaACLB2* (H) during leaf development. LS1, young leaves, 1 cm; LS2, growth leaves, 3 cm; LS3, mature leaves, 4 cm; LS4, old leaves. (I-L) Expression of *PaACLA1* (I), *PaACLA2* (J), *PaACLB1* (K) and *PaACLB2* (L) during flower development. S1-S6, six flower development stages. FS1 (0.5 cm in length), FS2 (1.0 cm), FS3 (2.0 cm), FS4 (3.0 cm), FS5 (4.0 cm) and FS6 (anthesis). *Actin* (accession no. FN014209) was used as the internal reference gene to quantify the cDNA abundance. Data are presented as the means ± SD (n = 3). Statistical analysis was performed using one-way analysis of variance (ANOVA) followed by Duncan's multiple range test (DMRT) with 3 biological replicates.

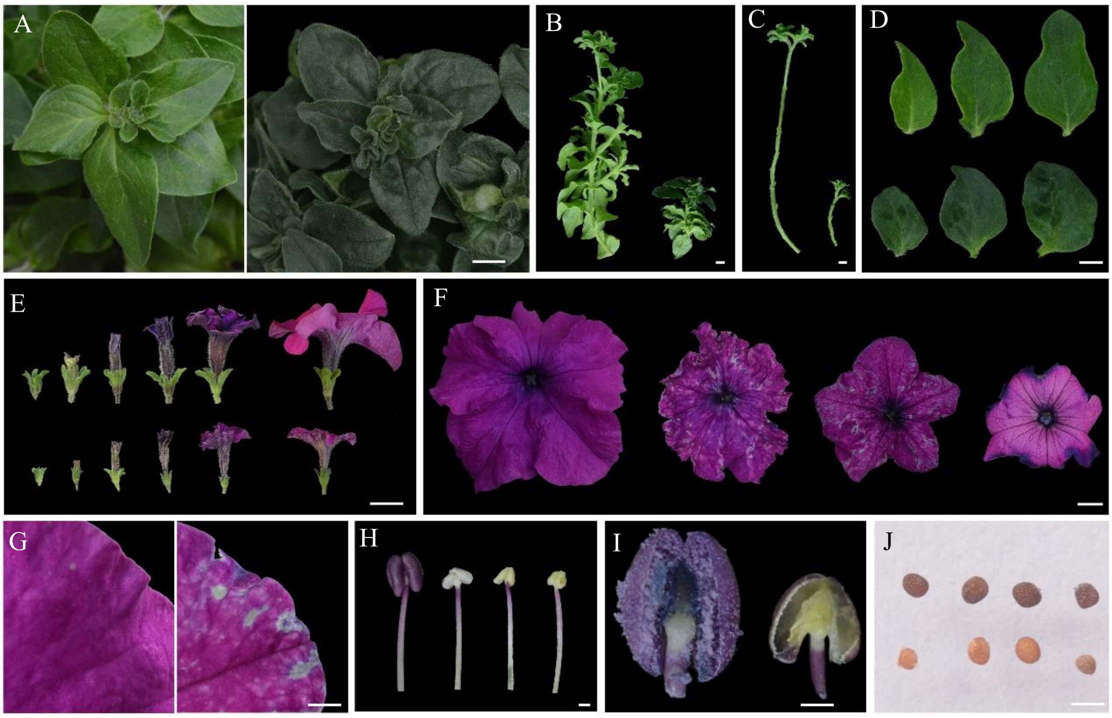

Figure S3 Phenotypical alteration of VIGS-mediated silencing of *PaACLB1* and *PaACLB2* in plants. (A-C) pTRV2- (left) and pTRV2-PaACLB1-B2-treated plant (right) branches from five-week-old plants. (D) Abnormal leaves of pTRV2-PaACLB1-B2-treated plants (right) and controls (left). (E) The six developmental stages of petunia buds of pTRV2-PaACLB1-B2-treated plants (bottom) and control (top). (F) Abnormal flowers of pTRV2-PaACLB1-B2-treated plants (right three) and controls (left). (G) Petals with transparent spots of pTRV2-PaACLB1-B2-treated plants (right) and control (left). (H) Abnormal anthers of pTRV2-PaACLB1-B2-treated plants (right three) and controls (left). (I) The anthers without pollen of pTRV2-PaACLB1-B2-treated plants (right) and control (left). (J) The seeds from pTRV2-PaACLB1-B2-treated plants (bottom) and control (top). Bars=1 cm in (A-C) and (F); bars=0.5 cm in (D); bars=2 cm in (E); bars=0.3 cm in (G); bars= 0.1 mm in (H); bars= 0.05 mm in (I) and (J).

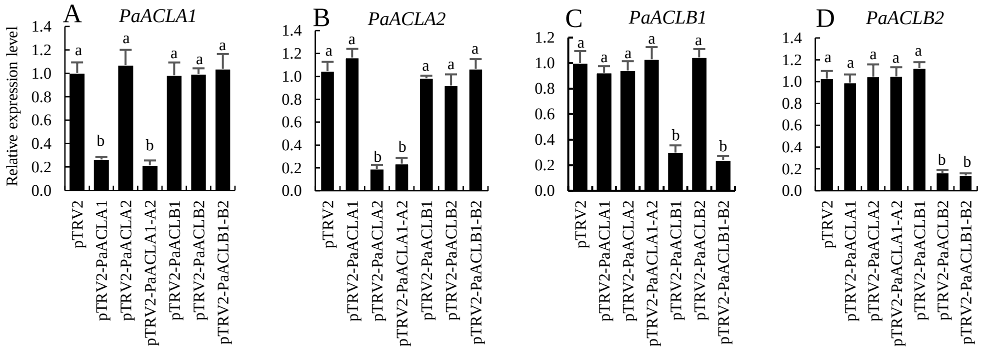

Figure S4 Expression of *PaACLAs* and *PaACLBs* determined using quantitative real-time PCR. (A-D) Expression of *PaACLA1* (A), *PaACLA2* (B), *PaACLB1* (C) and *PaACLB2*(D) in corollas of the control plants and *PaACLAs*- and *PaACLBs*-silenced plants. *Cyclophilin* (accession no. EST883944) was used as the internal reference gene to quantify the cDNA abundance. Data are presented as the means ± SD (n = 3). Statistical analysis was performed using one-way analysis of variance (ANOVA) followed by Duncan's multiple range test (DMRT) with 3 biological replicates. P-values ≤0.05 were considered significant.

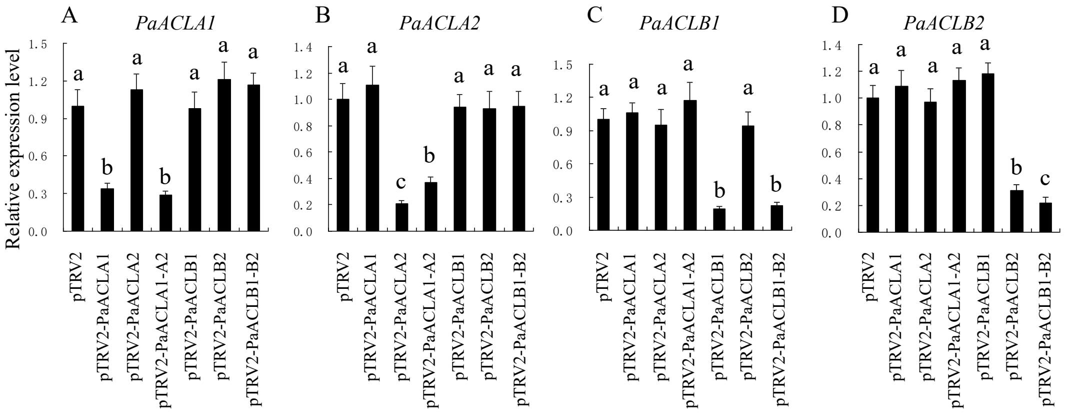

Figure S5 Expression of *PaACLAs* and *PaACLBs* determined by quantitative real-time PCR using *Actin* as the internal reference gene. (A-D) Expression of *PaACLA1* (A), *PaACLA2* (B), *PaACLB1* (C) and *PaACLB2*(D) in corollas of the control plants and *PaACLAs*- and *PaACLBs*-silenced plants. *Actin* (accession no. FN014209) was used as the internal reference gene to quantify the cDNA abundance. Data are presented as the means ± SD (n = 3). Statistical analysis was performed using one-way analysis of variance (ANOVA) followed by Duncan's multiple range test (DMRT) with 3 biological replicates. P-values ≤0.05 were considered significant.

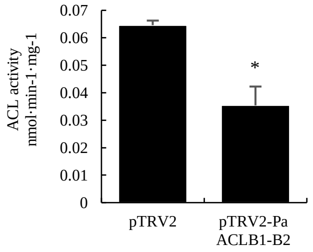

Figure S6 ACL activities of *PaACLB1-B2* silencing flowers and control. The data represents the mean and SD of three separate extractions.

A

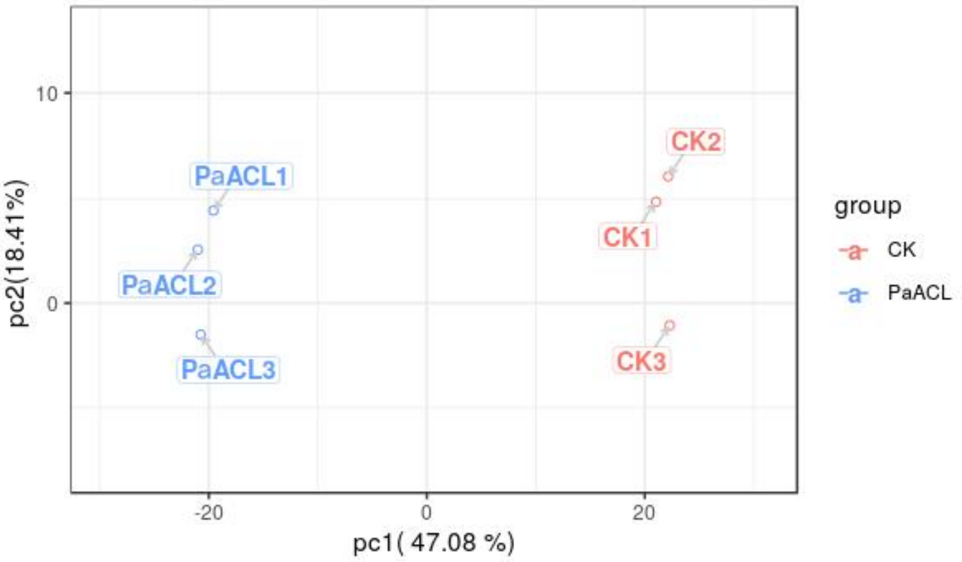

B

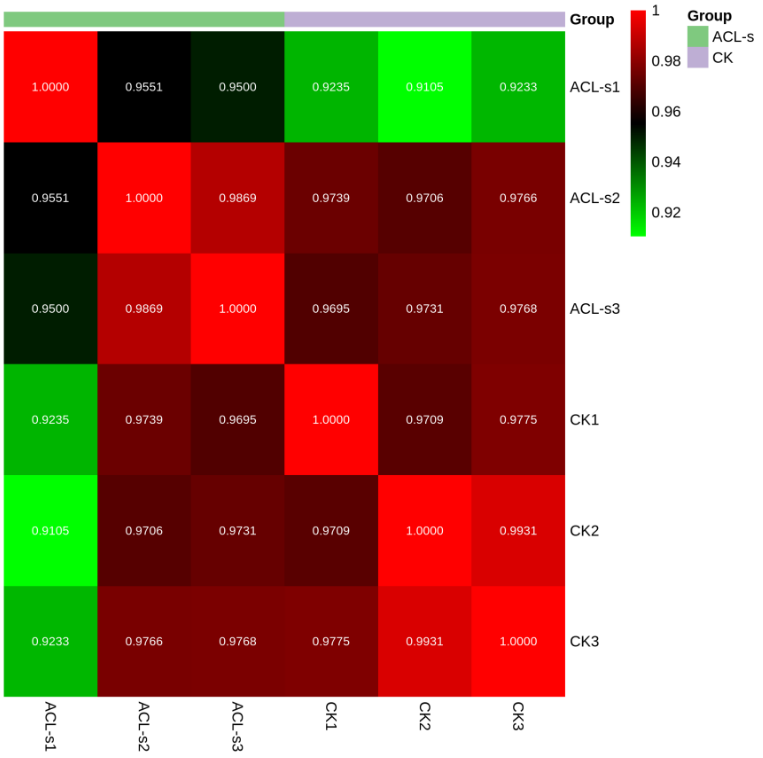

Figure S7 Repeatability analysis of metabolome and transcriptome. A, PCA score of mass spectrum data of samples and quality control samples in the metabolome. B, Repeatability test between samples in the transcriptome.

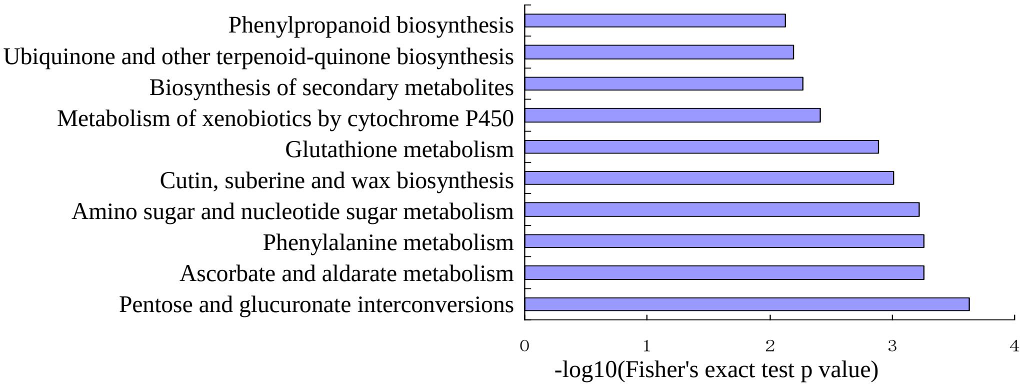

Figure S8 KEGG pathway enrichment analysis of differentially expressed genes in petunia corollas of *PaACLB1-B2*-silenced plants and control. The significance level was set at P <0.05 (Fischer's exact test). The data come from Supplemental Data Table 6.

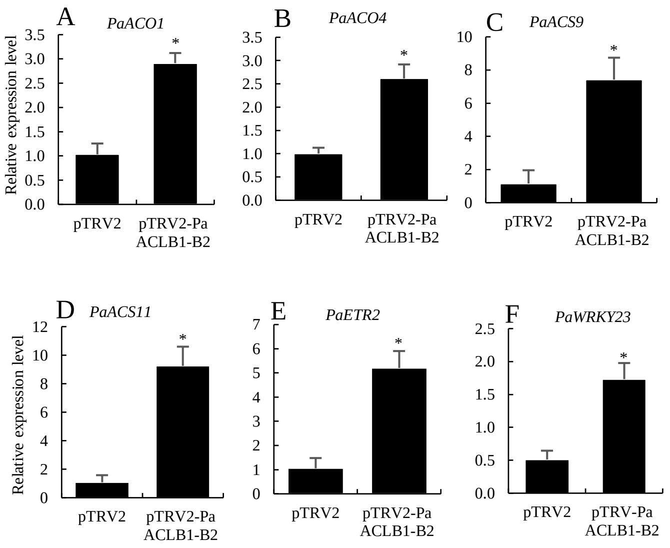

Figure S9 Confirmation of 6 senescence-related gene expression data by qPCR. Relative expression levels are shown as fold change values. *Cyclophilin* (accession no. EST883944) was used as the internal reference gene to quantify the cDNA abundance. Data are presented as the means ± SD (n = 3). Statistical analysis was performed using one-way analysis of variance (ANOVA) followed by Duncan's multiple range test (DMRT) with 3 biological replicates. P-values ≤0.05 were considered significant.

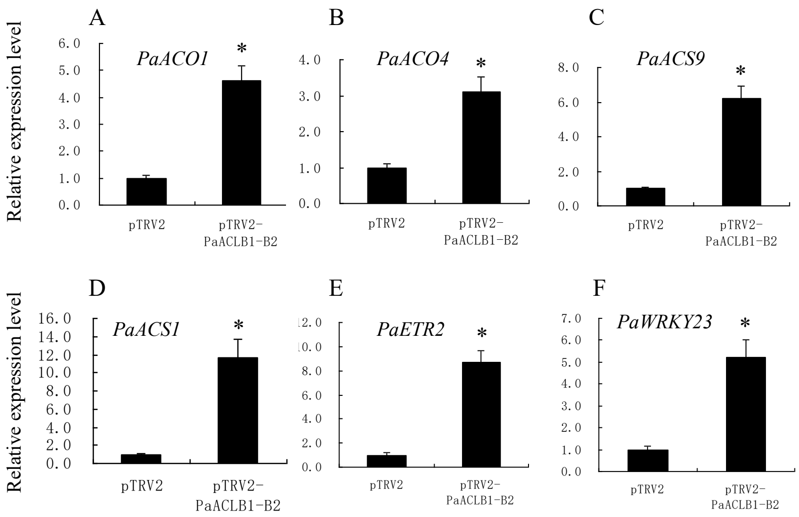

Figure S10 Confirmation of 6 senescence-related gene expression data by quantitative real-time PCR using *Actin* as the internal reference gene. Relative expression levels are shown as fold change values. *Actin* (accession no. FN014209) was used as the internal reference gene to quantify the cDNA abundance. Data are presented as the means ± SD (n = 3). Statistical analysis was performed using one-way analysis of variance (ANOVA) followed by Duncan's multiple range test (DMRT) with 3 biological replicates. P-values ≤0.05 were considered significant.

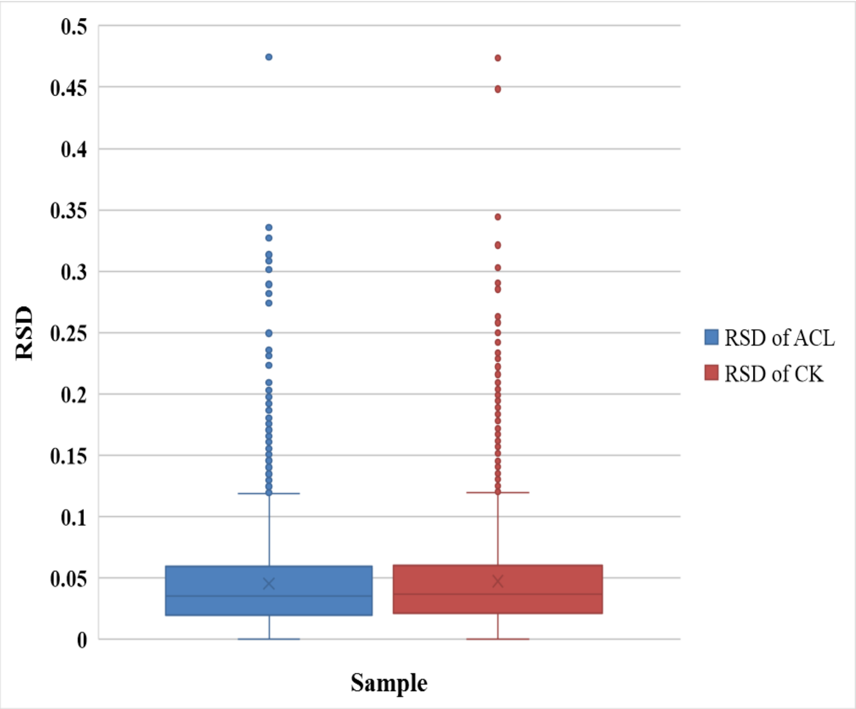

Figure S11 Repeatability test between samples in the proteome of petunia corollas of *PaACLB1-B2*-silenced plants and control.

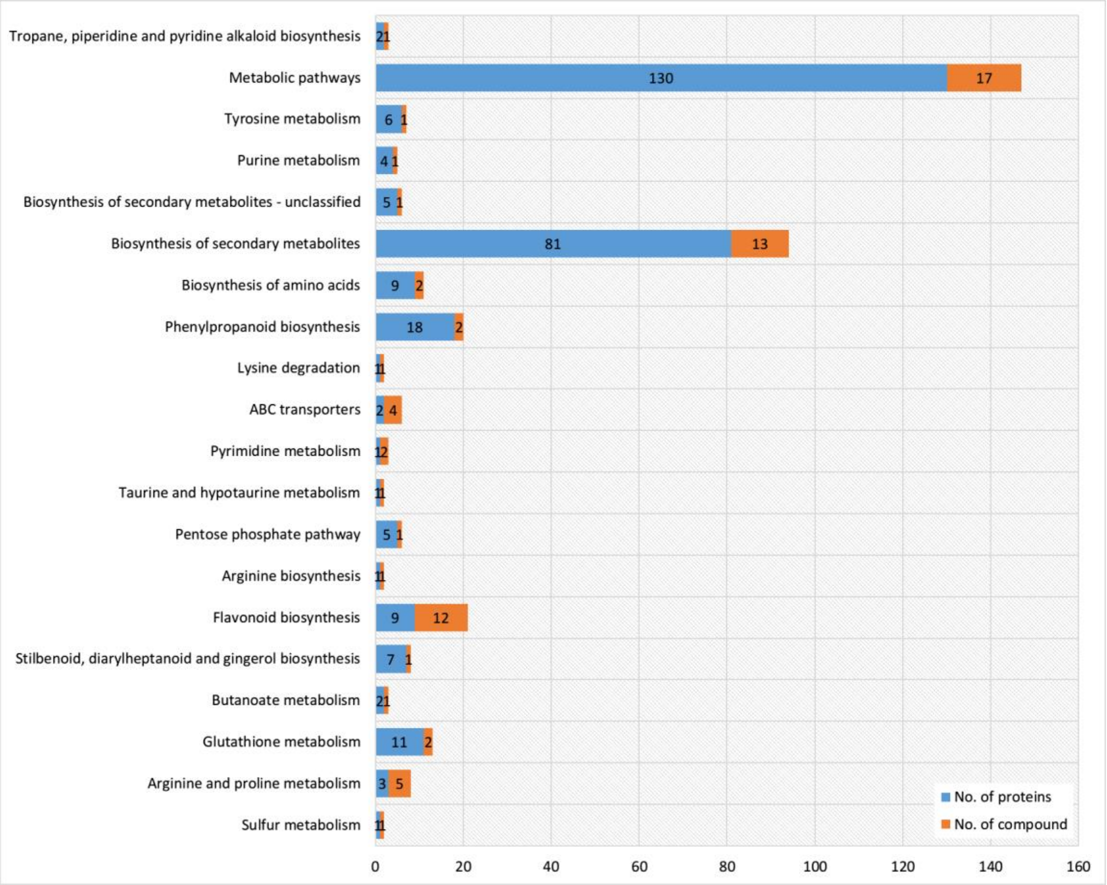

Figure S12 Distribution of KEGG pathways of differential proteins and differential metabolites.

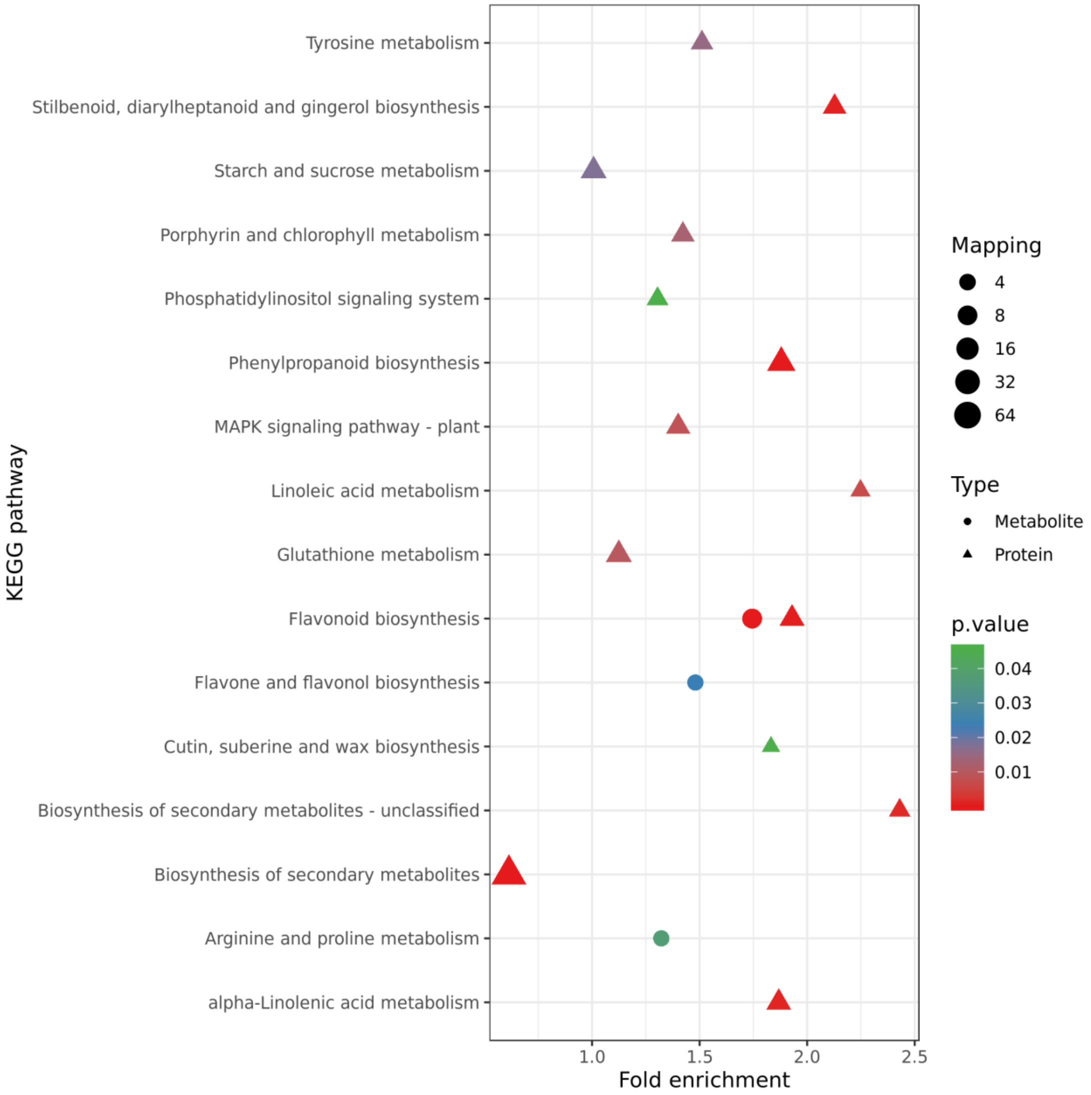

Figure S13 The bubble chart of KEGG enrichment analysis. The abscissa is the ratio of differential metabolites/proteins in the pathway to the background metabolite/protein identified in the pathway, and the ordinate is the corresponding entry in the KEGG. The triangle represents the metabolite, and the circle represents the protein. Shape size indicates the number of proteins or metabolites in the pathway. The shape colour indicates the significance of channel enrichment.

A

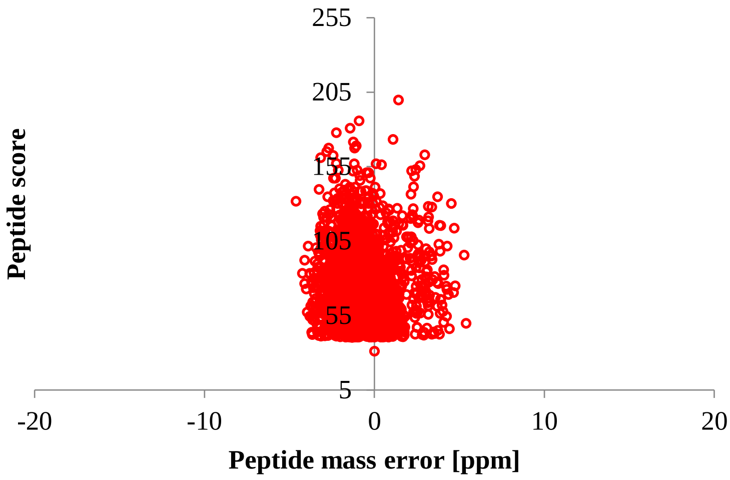

B

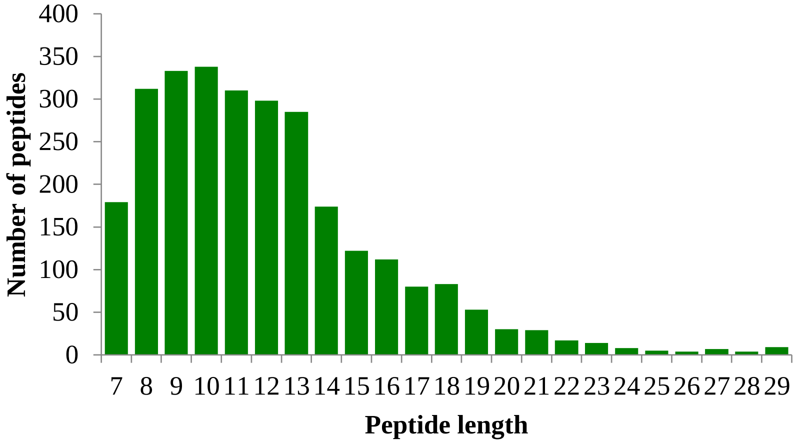

Figure S14 Quality control of mass spectrometry data. (A) The mass shift distribution of the identified peptide segment; (B) the length distribution of the identified peptide segment.

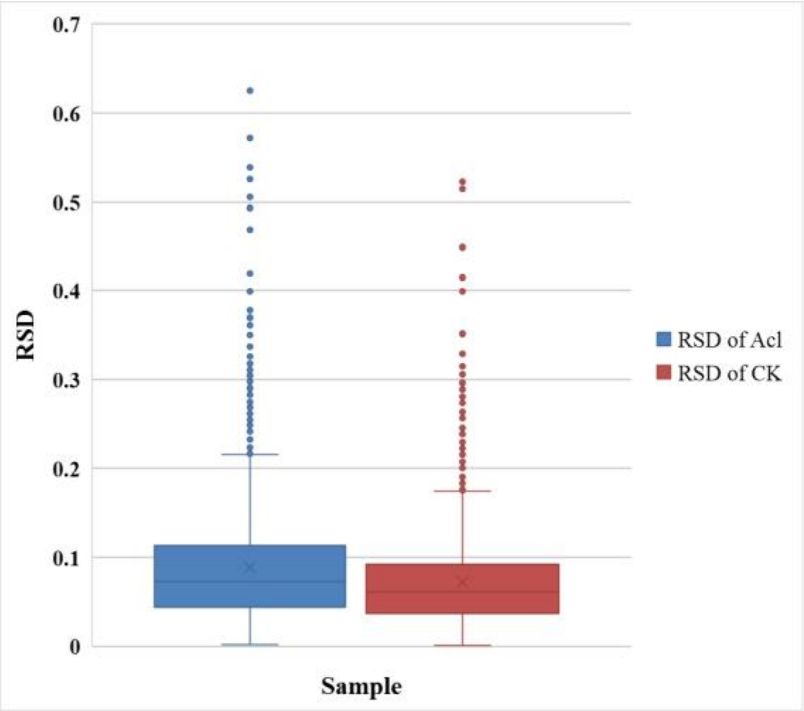

Figure S15 Repeatability test between samples in protein lysine acetylome.

A

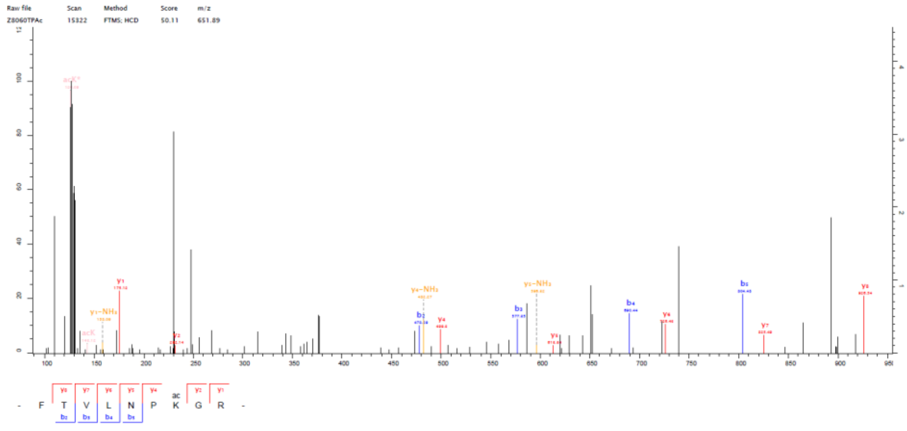

B

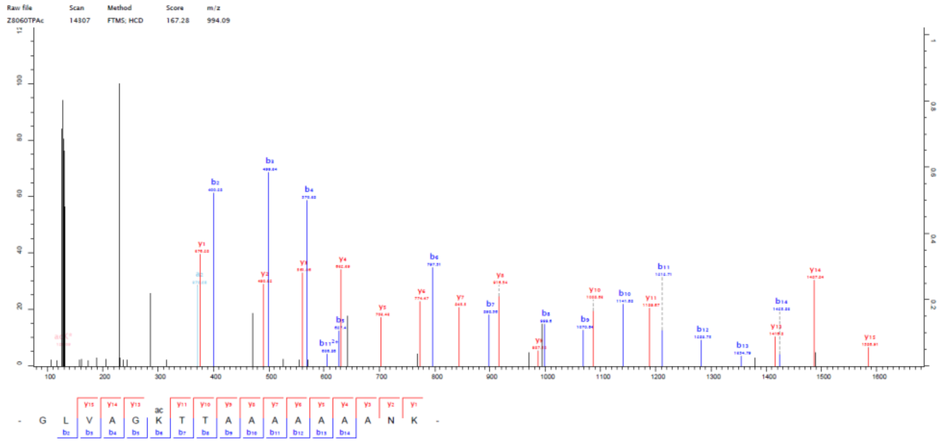

C

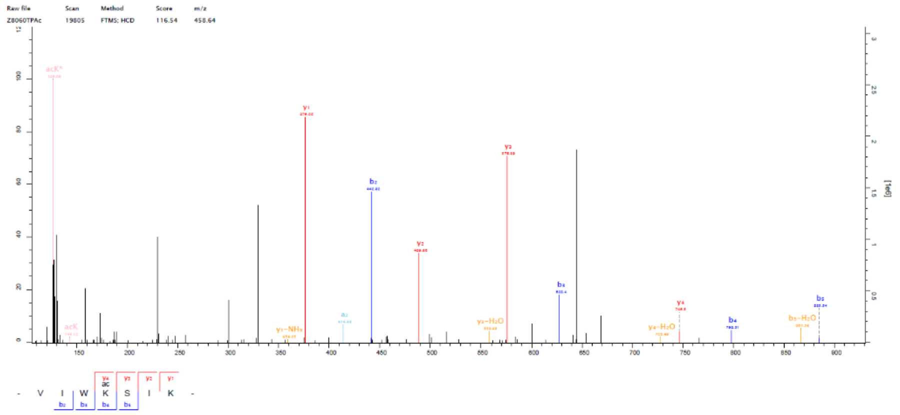

Figure S16 MS/MS spectra of lysine acetylation of several proteins. A, ATP-citrate lyase PaACLA1_269Kac (Peaxi162Scf00357g00428); B, isocitrate dehydrogenase IDH26Kac (Peaxi162Scf00705g00316); C, histone H2A.11_13Kac (Peaxi162Scf00608g00918).

A

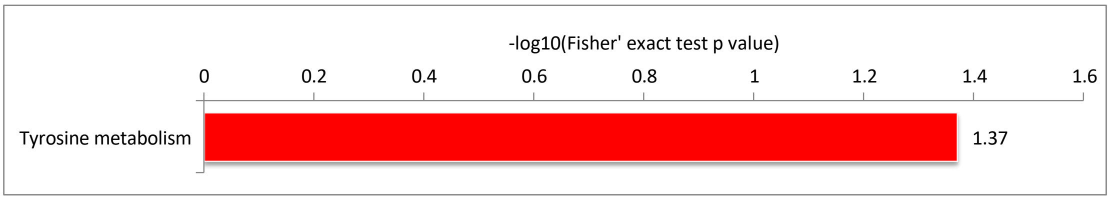

B

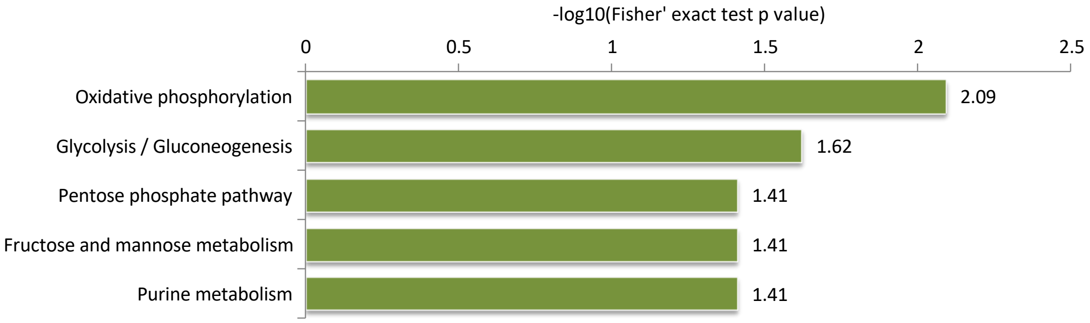

Figure S17 KEGG pathway enrichment analysis of proteins with upregulated and downregulated Kac sites. KEGG pathway enrichment analysis of proteins with upregulated (A) and downregulated (B) Kac sites. The significance level was set at P <0.05 (Fischer's exact test). The data come from Supplemental Data Table 9.

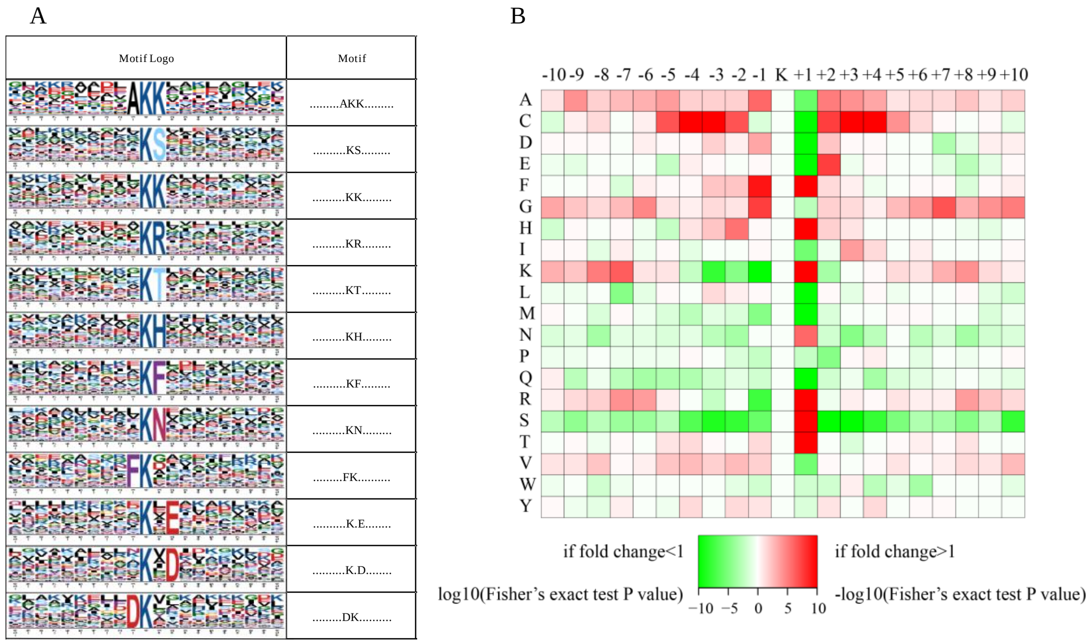

Figure S18 Motif analysis of all the identified Kac sites in petunia. A, Acetylation motifs and the conservation of Kac sites. The height of each letter corresponds to the frequency of that amino acid residue in that position. The central K refers to the acetylated lysine. B, Amino acid sequence properties of ubiquitylation sites. The heat map shows significant position-specific under- or overrepresentation of amino acids flanking the modification sites.

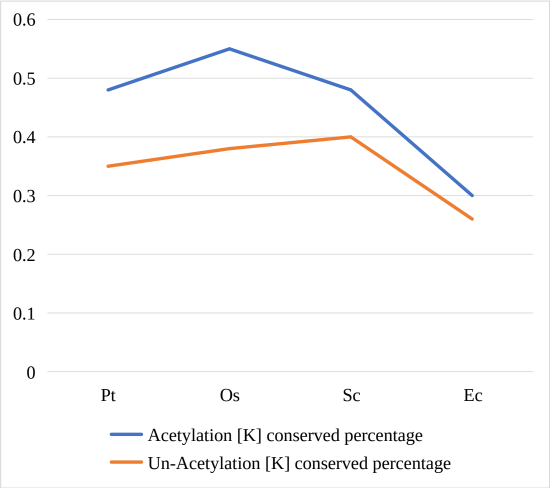

Figure S19 Evolutionary conservation of acetylated and non-acetylated lysines on protein orthologues in selected species. Abbreviations: Ec, *Escherichia coli*; Os, *Oryza sativa* japonica; Pt, *Phaeodactylum tricornutum*; Sc, *Saccharomyces cerevisiae*.

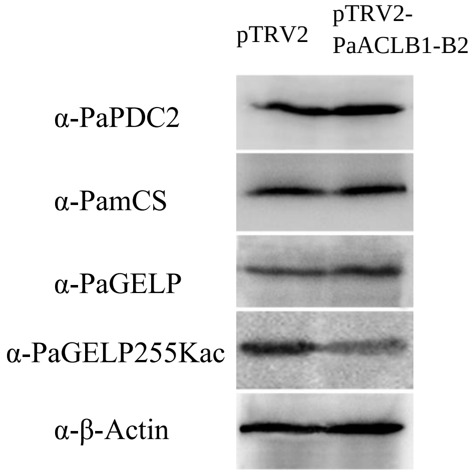

Figure S20 Confirmation of proteome (the first three) and acetylome data (the fourth one). Western blot analysis of petunia corollas treatment with pTRV2 and pTRV2-PaACLB1-B2. Representative results from three independent experiments are indicated.

A

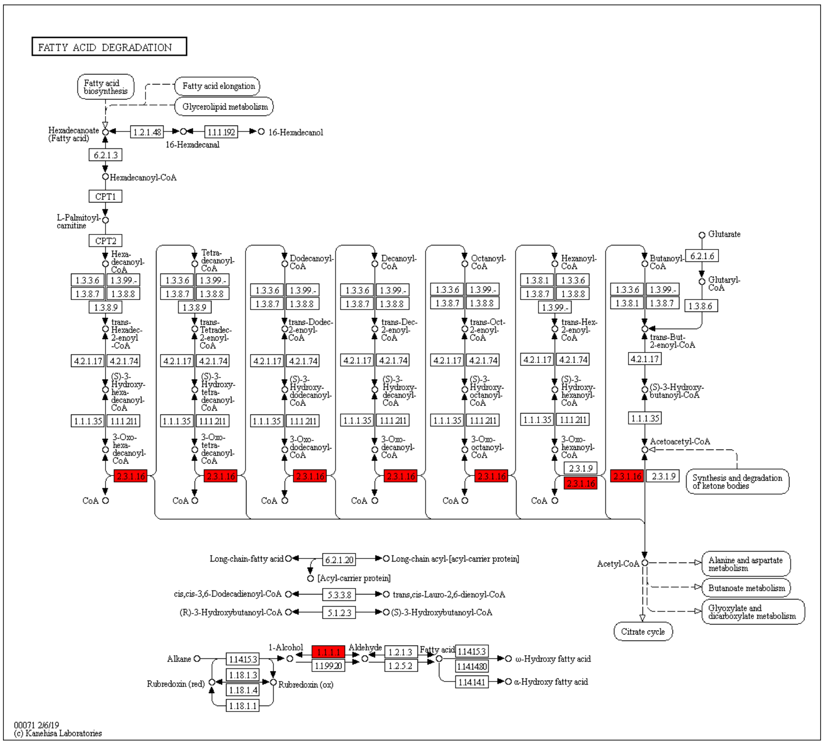

B

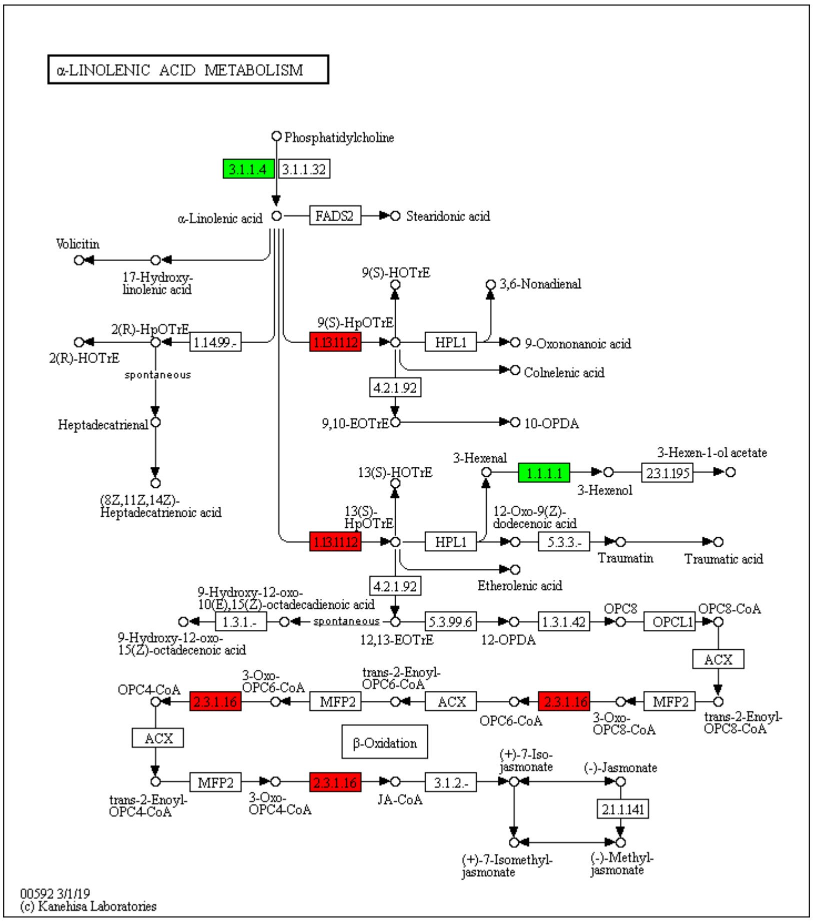

Figure S21 Differential proteins with acetylation sites in the opposite direction were enriched in the fatty acid degradation (A) and alpha-linolenic acid metabolism (B) KEGG pathways. Red box

indicates up-regulation and green box indicates down-regulation in acetylation level based on the statistical significance in this study.

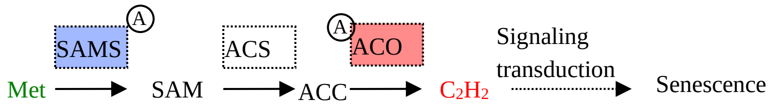

Figure S22 Effects of *PaACLB1-B2* silencing on the proteins engaged in ethylene biosynthesis in petunia. Differentially expressed proteins based on statistical significance in this study are framed in boxes, and differentially acetylated proteins have round boxes. The red box indicates upregulation; the green box indicates downregulation; and the blue indicates no significant changes upon pTRV2-PaACLB1-B2 treatment. Abbreviations: A, acetylation; ACC, 1-aminocyclopropane-1-carboxylic acid; ACO, ACC oxidase; ACS, ACC synthase; Met, methionine; SAM, S-adenosylmethionine; SAMS, S-AdoMet synthetase.

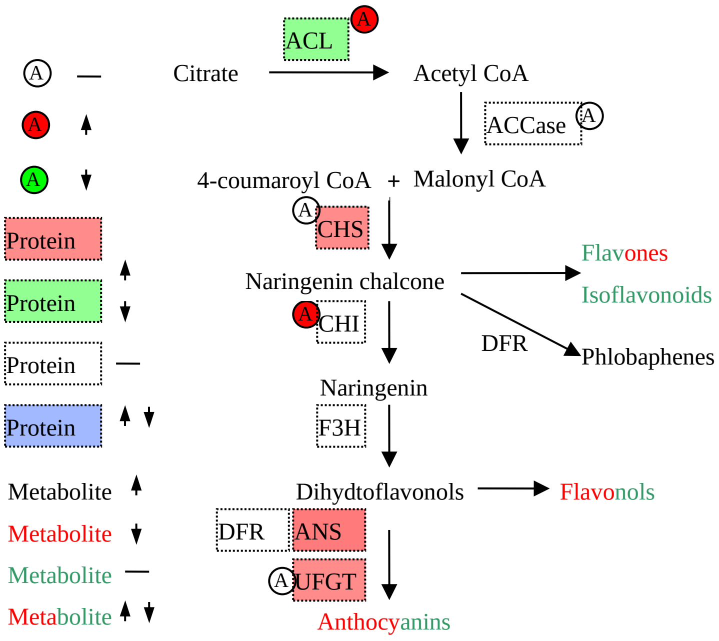

Figure S23 Effects of *PaACLB1-B2* silencing on anthocyanin and other flavonoid biosynthesis and the proteins engaged in their biosynthesis pathway in petunia. Differentially expressed proteins based on statistical significance in this study are framed in boxes, and differentially acetylated proteins are framed with circles. The red box indicates upregulation; the green box indicates downregulation; and the blue indicates no significant changes upon pTRV2-PaACLB1-B2 treatment. Abbreviations: 3GT, UDP-glucose:flavonoid 3-O-glycosyl transferase; 4CL, 4-coumarate:CoA ligase; A, acetylation;; ACCase, acetyl-CoA carboxylase; ANS, anthocyanin synthase; C4H, cinnamate-4-hydroxylase; CHS, chalcone synthase; CHI, chalcone flavanone isomerase; DFR, dihydroflavonol 4-reductase; F3H, flavanone 3ß-hydroxylase.
