# Supplementary material for: PaACL silencing accelerates flower senescence and changes the proteome to maintain metabolic homeostasis in Petunia hybrida
Source: J Exp Bot. 2020 May 4;71(16):4858–76. doi: 10.1093/jxb/eraa208 (PMC7475263; doi:10.1093/jxb/eraa208)
Supplement: eraa208_suppl_Supplementary_Tables_S1-S5 [file eraa208_suppl_supplementary_tables_s1-s5.pdf]

Table S1 Specific primer sequences used for full-length ACL gene isolation from *Petunia hybrida* 'Ultra'

Gene	Forward primer (5′→3′)	Reverse primer (5′→3′)
PaACLA1	ATGGCCAGAAAGAAGATCAGAGAG	TTATGCAGCTGCAGTGATGCACTC
PaACLA2	ATGGCAAGGAAGAAGATCAGAGAG	CTATGCTTCAGACATAATGCAATC
PaACLB1	ATGGCTACTGGACAACTTTTCTC	TCACTTGGTGTAGAGGACATCTT
PaACLB2	ATGGCTACTGGACAGCTTTTCTC	TCACTTGGTGTAGAGAACGTCTTC

Table S2 Primer sequences used in quantitative real-time PCR

Gene	Forward primer (5′→3′)	Reverse primer (5′→3′)
PaETR2	GCTTTGTTATCCGTTGCTGA	ATAATTCTCGGGATGTTCCA
PaACO1	TGAATGGTGCTGAAAGAG	CATTACTTCATGTGGGATT
PaACO4	CCCAAACTGACGGGACTC	AAGCTGGTGCAGGATAAA
PaACS9	CTGGTTTCGTGTATGTTT	TGGCGTTACTTTGCTGAT
PaACS11	GAAGCAGAAGCCAACAAC	CAAGAATACCCTCAAGGA
PaWRKY23	TGAATACGCCATCAACAC	CCAGCTACTACTTCTCCCT
PaACLA1	AAACGCCTTGCTGCCATTG	CCACTCTTTCCACGCTTCCC
PaACLA2	TGTTATTTGGGAAGCGTGGA	TCCGCCCATCTCCACCTC
PaACLB1	CTGATTTTACGAGGAGGGACAC	CTTAGTATTTGTTAGTGTGTGTAGTAATGGAA
PaACLB2	TTCTCCAACAACCACCTAACTG	TTTGTGTTGCTTTGATGAAATAGAG
CYP	AGGCTCATCATTCCACCGTGT	TCATCTGCGAACTTAGCACCG
Actin	TGCTGATCGAATGAGCAAGGAA	GGAGCAACAACCTTAATCTTC

Table S3 Specific primer sequences used for VIGS vector construction

Gene	Forward primer (5′→3′)	Reverse primer (5′→3′)
PaACLA1	ACGCGTGAGCTCGGTACCGGATCCCTCCGTTGATTGCTACTCTG	TCTGTGAGTAAGGTTACCGAATTCAGAACCTCCTCTTCATTTGG
PaACLA2	ACGCGTGAGCTCGGTACCGGATCCTCCCAATTGAGGTCTATGGAC	TCTGTGAGTAAGGTTACCGAATTCTGGACTTCATGCAGAAAGTGG
PaACLB1	ACGCGTGAGCTCGGTACCGGATCCAGCAGCCTTTGTACCGTCACCC	TCTGTGAGTAAGGTTACCGAATTCATCATAAATGATAGCTGAGAG
PaACLB2	ACGCGTGAGCTCGGTACCGGATCCATCCAGCATTTTATCCAAGTGT	TCTGTGAGTAAGGTTACCGAATTCAGCTTTAAACCAGGTACAACAA
PaACLA1-A2	ACGCGTGAGCTCGGTACCGGATCCTCTATGCTGATACAGTAGGTGA	GTGGCAACAAGTGATGTGCATTCTAGCTGCTTTT
	AGAATGCACATCACTTGTTGCCACTCTACCAT	TCTGTGAGTAAGGTTACCGAATTCCTCTGTAGCACTCATAACCCT
PaACLB1-B2	ACGCGTGAGCTCGGTACCGGATCCCGATTTGGTGGTGCTATTGATG	ATACCACCAGATATTTCTGGCTTGGTGAACATT
	ACGCGTGAGCTCGGTACCGGATCCTCTATGCTGATACAGTAGGTGA	GTGGCAACAAGTGATGTGCATTCTAGCTGCTTTT

Table S4 The peptides expressed in *Escherichia coli* of three proteins, PamCS, PaPDC2 and PaGELP

protein	Peptide sequence
PamCS	VPSKEQVDSLSQELQSRATIPDHVYKTIDALPVTAHPMTQFATGVMALQVQSEFQKAEKGIHKSKLWEPTYEDSMSLIAQVPLVGAYVYRRMYKNGQTIPKDDSLDYGANFAMLGYSSPDMHELMRLYVTIHSDHEGGNVSA
PaPDC2	NYTGLVDAIHNGEGNCWTTKVRCEEELVEAIETATGAKKDSLCFIEVIVHKDDTSKELLEWGSRVSAANSRPPNPHFWFESHEGSIDTCKPTTNNVATLPTNNTVTISTHTPSTTIIPCNSTTDST
PaGELP	QDSTTLVPAIITFGDSAVDVGNNDYIHTLFKANYPPYGRDFVSHQPTGRFCNGKLATDITADTLGFTTYPAAYLSPQASGKNLLIGTNFASAAAGYDDKTAILNHAIPLSQQMQFYKEYQSKLAKVAGSQKATSILKDALYIISAGSSDFLQNYYINPYINKIYTPDQYG

Table S5 Effects of *PaACLB1-B2* silencing on the plants and cell size

	pTRV2	pTRV2-PaB1-B2
height of plant (cm)	18.53 ± 1.01	12.45 ± 1.55*
Diameter of plant (cm)	18.95 ± 0.97	13.71 ± 0.89*
height of Internode (cm)	2.55 ± 0.57	0.51 ± 0.11*
Length of young leaves (cm)	2.13 ± 0.05	1.33 ± 0.05*
Length of young leaves (cm)	2.13 ± 0.05	1.33 ± 0.05*
Length of pedicel (cm)	2.86 ± 0.41	0.76 ± 0.09*
Length of sepal (cm)	2.35± 0.17	1.12 ± 0.15*
Diameter of corolla (cm)	7.85± 0.54	5.45 ± 0.62*
Length of filaments (cm)	3.86 ± 0.41	3.12 ± 0.15*
Length of styles (cm)	3.85 ± 0.06	3.09 ± 0.19*
Diameter of stigma (mm)	3.13 ± 0.05	1.95 ± 0.06*
Diameter of leaves upper epidermal cells (μm)	41.22±1.83	20.18 ± 1.14*
Diameter of leaves lower epidermal cells (μm)	42.84±1.21	19.66 ± 1.17*
Length of guard cells (μm)	20.51±1.33	16.53 ± 1.61*
Diameter of petals upper epidermal cells (μm)	2.50 ± 0.11	1.58 ± 0.09*
Diameter of petals lower epidermal cells (μm)	2.81 ± 0.12	2.24 ± 0.10 *
Wall thickness of mesophyll cells	0.30 ± 0.03	0.46 ± 0.02*
Wall thickness of floral cells	0.24 ± 0.02	0.59 ± 0.03*
Wall thickness of flower adaxial epidermis cells	1.8 ± 0.02	2.61 ± 0.02*

* Data are means ± SE from 15 to 20 samples. Statistical analysis was performed using Student t test with 15 to 20 replicates. Asterisk means significant difference at P=0. 05 level.
